# Global stratigraphic dataset composing LOK2016CS database: Biostratigraphic ranges of uppermost Tithonian-Hauterivian bioevents (Lower Cretaceous): Tables 1 and 2

**DOI:** 10.1016/j.dib.2019.104244

**Published:** 2019-08-05

**Authors:** Robert W. Scott

**Affiliations:** Precision Stratigraphy Associates, Cleveland, OK, USA

**Keywords:** Jurassic-Cretaceous boundary, Numerical ages of bioevents, Magnetozones

## Abstract

The Research article “Jurassic-Cretaceous boundary bioevents and magnetochrons: A stratigraphic experiment” (Scott, 2019), describes the Lower Cretaceous 2016 Chronostratigraphic Database (LOK2016CS). This database comprises the first and last occurrences of key fossil ranges in forty-one global stratigraphic sections (Table 1). This data has been published by qualified biostratigraphers in selected worldwide sections that have been carefully measured, collected and fossil species consistently identified. Regional reference sections and approved or proposed Global Boundary Stratotype Section and Point (GSSP) are included. This data set is composed of lowermost and uppermost positions of species measured in meters or feet by the research team in each section, which represent the first and last occurrences of the species. These data sets were composited into the numerical age chart, LOK2016CS (Table 2), by graphic correlation. The 2016 Geologic Time Scale was the X-axis section on the first X/Y plot so that the numerical ages were calibrated consistently with that scale. The numerical ages of each event estimate the First and Last Appearance Datums (FAD, LAD) within the geographic area encompassed by the sampled sections.

**Table:** Specifications table

Subject area	*Geosciences*
More specific subject area	*Biostratigraphy*
Type of data	*Accurate, precise geographic locations of 41 worldwide sections given in*Table 1*. Raw data are Lists of fossil species and magnetozones and the lowest and highest positions in numerous measured sections in the Tethyan Realm in*Table 1*. Analyzed/processed Numerical ages of bioevents and magnetozones in*Table 2*.*
How data was acquired	*Fossils were identified by numerous researchers using microscopes specific to their labs; more detail is in each reference.*
Data format	*Raw data of published lowest and highest positions in meters or feet*Table 2*.*
Experimental factors	*Fossil records are subject to sedimentological processes, to collection density, to preservation, and to the skill of the operators.*
Experimental features	*Fossil FO and LO events were projected onto the X-axis by a correlation line determined by the experimenter and numerical ages were interpolated; by plotting multiple sections maximum ranges were estimated.*
Data source location	*The geographic location of each data section is given in*Table 1*.*
Data accessibility	*Data of each section has been published by the original authors and the references are in the section descriptions (*Table 1*).*
Related Research Article	R.W. Scott, Jurassic–Cretaceous boundary bioevents and magnetochrons: A stratigraphic experiment, Cret. Res., 100 (2019) 97–104. 10.1016/j.cretres.2019.03.007[1].

**Table:** 

**Value of the data** • This data provides precise numerical ages of latest Tithonian to Hauterivian stage marine fossils • This data benefits geoscientists needing precise ages to test geoscientific hypotheses • Data can be used to scale timing of geological events • Data can be used to estimate rates of evolution and timing of biogeographic migrations • Data can be used to experiment with correlating stratigraphic sections in a time frame

## 1 Data

The raw data presented below (Table 1) consists of species reported in forty-one well documented sedimentary sections in many parts of the world and their lowest/oldest and highest/youngest positions in these sections. The tops of magnetozones are also listed. Each section is named and has a computer file name: LOC.X. The locations are given in longitude and latitude and reference(s) to original data are provided for each section. Table 2 is a list of the species and their FAD/LAD ages analyzed by graphic correlation. Numerical ages of stratigraphic sections also determine durations of source rock maturation to identify types of hydrocarbons. Also, numerical ages are used in estimating rates and durations of tectonic and igneous processes.

The data is in table form to serve as a look-up list so that geoscientists who find any of these species in their research may use these ages to calibrate the age of the sedimentary units, to correlate several stratigraphic sections, to calculate rates of sediment accumulation, to estimate the durations of hiatuses, and to measure the rate of evolution of phylogenetic clades, diversification and migration.

## 2 Experimental design, materials and methods

The Table 1 lists the raw data of species and magnetozones in each of the forty-one sections used to compile the analyzed numerical ages of the chronostratigraphic events in LOK2016CS database (Table 2). The fossils in each section were processed from the sedimentary rocks by standard methods and were identified by standard microscopic and scanning electron microscopic methods. References of each section provides details of geographic location, sample processing and identifications.

These data sets were composited into the numerical age chart, LOK2016CS (Table 2), by **graphic correlation** [2], [3] using the GraphCor software [4]. The 2016 Geologic Time Scale was the X-axis section on the first X/Y plot so that the numerical ages were calibrated consistently with that scale [1]. The numerical ages of each event estimate the First and Last Appearance Datums (FAD, LAD) within the geographic area encompassed by the sampled sections.

Graphic correlation is a quantitative, non-statistical, technique that determines the coeval relationships between two sections by comparing the ranges of event records in both sections [2]. A graph of any pair of sections is an X/Y plot of the FOs (first appearances) and LOs (last appearances) of taxa found in both sections. The interpreter places a line of correlation (LOC) through the tops and bases that are at their maximum range in both sections. This LOC is the most constrained hypothesis of synchroneity between the two sections and extends the fewest bioevents. The LOC also accounts for hiatuses or faults at stratal discontinuities indicated by the lithostratigraphic record. The position of the LOC is defined by the equation for a regression line. Explanation and examples of the graphic technique are illustrated by [2]. By iteratively graphing successive sections a database of species ranges is compiled. The accuracy of these ranges depends on the number of sections, preservation and correct identification of the species. The succession of events already scaled in mega-annums as the standard section, such as the 2016 Geologic Time Scale, results in a time scale of composited events. Such a database can be tested, and the process is transparent so that fossil occurrences in each section can be evaluated to determine their accuracy. This process integrates data from numerous global sections analyzed by numerous specialists.
