# Supplementary material for: Global stratigraphic dataset composing LOK2016CS database: Biostratigraphic ranges of uppermost Tithonian-Hauterivian bioevents (Lower Cretaceous): Tables 1 and 2
Source: Data Brief. 2019 Aug 5;25:104244. doi: 10.1016/j.dib.2019.104244 (PMC6699454; doi:10.1016/j.dib.2019.104244)
Supplement: Supplementary file 1 [file mmc1.docx]

**Acknowledgements**

The skill and effort of many biostratigraphers have documented and published their data in the form of range charts. This experimental graphic method was developed by Allan Shaw [5] in the 1960’s and was extensively applied by Amoco Research Center Biostratigraphy Group in the 1980’s. Many Amoco stratigraphers have constructively influenced my understanding and appreciation of biostratigraphic data and its utility in unraveling Earth History and supporting resource exploration and development.

**References**

[1] R.W. Scott, Jurassic–Cretaceous boundary bioevents and magnetochrons: A stratigraphic experiment, Cret. Res., 100 (2019) 97–104. [10.1016/j.cretres.2019.03.007](https://doi.org/10.1016/j.cretres.2019.03.007).

[2] J.L.Carney, R.W. Pierce, Graphic correlation and composite standard databases as tools for the exploration biostratigrapher, SEPM Soc. for Sediment., Sp. Publ. No. 53 (1995) 23–43.

[3] L.E. Edwards, Graphic correlation: Some guidelines on theory and practice and how they relate to reality. Soc. for Sediment., Sp. Publ. No. 53 (1995) 45–50.

[4] K.C.Hood, GraphCor: Interactive Graphic Correlation Software, Version 3.0, Published by K.C. Hood, 9707 (1996) Houston, TX 77064.

[5] A.B.Shaw, Time in stratigraphy, McGraw-Hill, New York (1964) 365 p.

**Table1. Sections and rawstratigraphic data of each that comprise LOK2016CS Database.**

Morphology codes identify fossil groups: AM - ammonites; BI – bivalves; CA – calpionellids; DN – dinoflagellate; FB – benthic foraminifers; FP – planktic foraminifers; GC – geochemical signature; ID – indeterminate; MA – magnetochrons; MB – chronostratigraphic unit; NN – nannofossils; triple asteriks, ***, indicate absence of data.

CRET.16B 2016 Geologic Time Scale J/K to base Albian;

Ogg, J.G., Ogg, G.M., Gradstein, F.M., 2016, A concise geologic time scale 2016. Elsevier, Amsterdam, Netherlands, 234 p. **Magnetochrons.**

Base Albian /MB -113.14 ***

Microhedbergellarenilaevis /FP -113.14 -113.14

Leymeriellaschrammeni anterior /AM -113.1 -113.1

Prediscosphaeracolumnata /NN -113.1 -113.1

Wollemanniceraskeilhackianterior /AM -113.1 -113.1

Wollemanniceraskeilhackikeilhacki /AM -113.1 -113.1

Base Aptian /MB -126.3 ***

Deshayesites oglanlensis /AM -126.3 -126.3

Magnetochron CM0R /MB -126.3 ***

Base Barremian /MB -130.8 ***

Spitidiscus hugii /AM -130.8 -130.8

Base Hauterivian /MB -134.7 ***

Acanthodiscus radiatus /AM -134.7 -134.7

Base Valanginian /MB -139.4 ***

Calpionellites darderi /CA -139.4 -139.4

Thurmanniceraspertransiens /AM -139.4 -139.4

Calcicalathina oblongata /NN -139 -133

Speetonia colligata /NN -139 -133

Base Berriasian /MB -145.5 ***

Calpionella alpina /CA -145.7 -145.7

*Ages in Ogg et al. 2012 Time Scale interpolated into 2016 fig. 13.4 given as age of base, here; re-assigned to top of previous chron to coincide with depths of tops

Magnetochron M15n /ma *** -139.6

Magnetochron M17n /ma *** -142.5

Magnetochron M18n /ma *** -144.0

Magnetochron M19n /ma *** -145.0

LOK.1 DSDP 534 Blake Plateau Offshore Florida, 28° 20.6'N, 75° 22.9'W;

Sheridan, R. E., et al., 1983, Initial Reports DSDP 76, Washington, U.S. Govt. Printing Office; Bergen, J.A., 1994, Journal Nannoplankton Research, 16:59-69. **Magnetochrons**from Ogg, 1983, DSDP 76 Init.Repts., p. 685-697.

Magnetochron M16n /ma * -1264.2

Magnetochron M16r /ma * -1269.8

Magnetochron M17n /ma * -1277.0

Magnetochron M17r /ma * -1283.9

Magnetochron M18n /ma * -1314.3

Magnetochron M18r /ma * -1323.9

Magnetochron M19n /ma * -1329.2

Magnetochron M19r /ma * -1357.0

Magnetochron M20n /ma * -1365.6

Magnetochron M20r /ma * -1374.0

Remane, 1983, DSDP 76, p. 561-566; zone boundaries not definable

Calpionella alpina /ca -1350.3 -1307.0

Crassicollaria intermedia /ca -1350.3 -1350.1

Crassicollariamassutiniana /ca -1348.0 -1348.0

Crassicollariaparvula /ca -1352.9 -1326.7

Tintinnopsellacarpathica /ca -1350.3 -1323.9

Roth, 1982, Init.Repts. DSDP, 76, p. 587ff; Bergen '94, J. Nannoplankton Res., 16:59-69; selected data only

Acaenolithusvimineus /nn -981.5 -963.0

Amphizygus brooksii /nn -1075.0 -979.0

Axopodorhabdusdietzmannii /nn -1202.0 -950.7

Biscutumconstans /nn -1305.5 -950.0

Braarudosphaeraregularis /nn -1047.6 -1047.6

Bukrylithus ambiguus /nn -1211.5 -950.7

Calcicalathina oblongata /nn -1239.5 -975.5

*Bergen '94; FO = base Val/ LO = mid Barr, Mutterlose, '92

Chiastozygusbilamellus /nn -1233.5 -1137.4

Chiastozygusstriatus /nn -1075.0 -972.5

Chiastozygus tenuis /nn -1224.5 -1143.6

Conusphaera mexicana /nn -1340.0 -950.0

Corollithiongeometricum /nn -1139.5 -974.5

Cretarhabdus conicus /nn -1287.5 -950.0

Cruciellipsiscuvillieri /nn -1268.0 -1020.5 *Bergen '94 top pick

Cruciplacolithusfurtivus /nn -1075.0 -1011.8

Cruciplacolithussalebrosus /nn -1264.0 -1196.4

Cyclagelosphaeradeflandrei /nn -1340.0 -1220.0

*Bergen '94 picked top; Roth picked top at 1130.0

Cyclagelosphaeramargerelii /nn -1340.0 -950.0

Diadorhombus rectus /nn -1222.5 -1026.8

Diazomatolithuslehmanii /nn -1325.6 -950.0

Dilomaplacinum /nn -1110.6 -1107.5

Discorhabdus ignotus /nn -1287.2 -950.0

Ethmorhabdushauterivianus /nn -975.5 -975.5 *Bergen '94

Gaarderellagranulifera /nn -965.7 -950.7

Grantarhabdusmeddii /nn -1170.8 -1056.8

Haqiuscircumradiatus /nn -1289.0 -950.0

*Bergen '94; Roth picked base at 1176.2

Hayesites radiatus /nn -1064.3 -953.1

Heleneachiastius /nn -1280.0 -950.0

Hexalithus noelae /nn -1340.0 -1331.2

Lithastrinusseptentrionalis /nn -1075.0 -950.0

Lithraphiditesbollii /nn -1119.5 -993.5

*Bergen '94; FO = mid Haut, Mutterlose, '92

Lithraphiditescarniolensis /nn -1334.1 -950.0

Manivitellapemmatoidea /nn -1277.0 -950.0

Micrantholithusobtusus /nn -1139.0 -972.7

Nannoconusbermudezii /nn -1206.8 -950.0

Nannoconus boletus /nn -1067.8 -1067.8

Nannoconus bonetii /nn -1107.8 -1107.8

Nannoconusbroennimannii /nn -1331.2 -1269.0

Nannoconusbucheri /nn -1137.6 -950.0

Nannoconus colomii /nn -1340.0 -950.0

Nannoconus cornutus /nn -1093.8 -1093.8

Nannoconus globulus /nn -1334.1 -950.0

Nannoconussteinmannii /nn -1332.5 -1332.5 *Bergen '94

Octopodorhabdusdecussatus /nn -1232.2 -1196.4

Octopodorhabduspolytretus /nn -1058.7 -1008.5

Octopodorhabdusreinhardtii /nn -1135.5 -1101.7

Parhabdolithusachlyostaurion /nn -1261.3 -1233.5

Parhabdolithusinfinitus /nn -1189.2 -950.0

Parhabdolithusswinnertonii /nn -1233.5 -1076.8

Polycostella senaria /nn -1305.6 -1305.6

Polypodorhabusmadingleyensis /nn -1337.4 -1058.8

Reinharditesfenestratus /nn -1268.8 -950.0

Retecapsaangustiforata /nn -1331.0 -950.0

*Bergen '94; Roth picked base at 1276.9

Retecapsa levis /nn -966.2 -960.9

Retecapsaneocomiana /nn -1292.0 -1089.8

Rhabdolekiskusparallelus /nn -1075.0 -966.2

Rhagodiscus asper /nn -1340.0 -950.0

Rhagodiscuseboracensis /nn -1148.8 -1148.8

Rhagodiscus splendens /nn -1289.9 -950.0

Rotelapilluslaffittei /nn -1289.6 -950.0

Rotelapillus radians /nn -1301.1 -1301.1

Rucinolithusirregularis /nn -981.5 -958.7

*FO = Barr/Apt, Mutterlose, '92

Rucinolithusterebrodentarius /nn -981.5 -981.5

*Bergen '94

Rucinolithuswisei /nn -1260.5 -1203.5

*Bergen '94

Sollasites horticus /nn -1268.8 -1196.4

Sollasites lowei /nn -972.5 -972.5

Speetonia colligata /nn -1271.8 -1008.5

*Bergen '94 picked top at 1001.0; LO = top Haut, Mutterlose, '92

Stradneria crenulata /nn -1296.8 -950.0

Tegumentum stradneri /nn -1058.7 -959.4

Tetrapodorhabduscoptensis /nn -1099.2 -959.4

Tranolithusgabalus /nn -951.7 -950.6

Tubodiscus verenae /nn -1229.5 -1107.5

Vagalapilla stradneri /nn -1287.5 -950.0

Vekshinella angusta /nn -1113.9 -992.5

Watznaueriabarnesae /nn -1340.0 -950.0

Watznaueria biporta /nn -1170.0 -950.7

Watznaueriabritannica /nn -1340.0 -950.0

Watznaueria communis /nn -1340.0 -950.0

Watznaueria oblonga /nn -1155.8 -954.5

Watznaueriasupraretacea /nn -1107.7 -951.7

Zeugrhabdotusembergeri /nn -1340.0 -950.0

Zygodiscusdiplogrammus /nn -1221.0 -950.0

Zygodiscus elegans /nn -1268.0 -950.0

Zygodiscus erectus /nn -1340.0 -1001.0 *Bergen '94 top pick

*Habib &Drugg, 1983, DSDP 76:623-635, Fig. 2

Achomosphaeraneptuni /dn -1235.3 -1113.9

Aldorfiadictyotum /dn -1176.3 -1176.3

Amphorulametaelliptica /dn -1283.5 -1243.1

Biorbiferajohnewingii /dn -1287.5 -1205.1

Callaiosphaeridiumasymmetricum /dn -1080.9 -950.0

Chlamydophorellamembranoidea /dn -1113. -1113.9

Chlamydophorellanyei /dn -975.0 -950.0

Circulodiniumdistinctum /dn -1277.0 -950.0

Cometodinium? whitei /dn -954.5 -954.5

Coronifera oceanica /dn -1066.3 -950.0

Crassiculosphaeridia reticulata /dn -975.0 -950.0

Cribroperidinumsepimentum /dn -1080.9 -950.0

Dapsilidiniumwarrenii /dn -1252.0 -966.8 *=Polysphaeridium

Dicanthumhollisteri /dn -1287.5 -950.0

Dingodiniumcerviculum /dn -1252.0 -950.0

Druggidiumapicopaucicum /dn -1243.1 -1080.9

Druggidium deflandrei /dn -1193.4 -950.0

Druggidiumrhabdoreticulatum /dn -1099.2 -950.0

Endoscrinium campanula /dn -1277.0 -950.0 *=Scrinodinium

Escharisphaeridiapocockii /dn -1287.5 -1287.5

Gonyaulacystahelicoidea /dn -1340 -950.0

Hystrichodiniumvoigtii /dn -1277.0 -966.8

Kiokansium polypes /dn -1243.1 -950.0 *=Bacchidinium

Muderongia simplex /dn -1283.4 -1243.1

Muderongia staurota /dn -1091.4 -966.8

Odontochitinaoperculata /dn -1080.9 -950.0

Oligosphaeridium complex /dn -1176.2 -950.0

Oligosphaeridiumpulcherrimum /dn -1205.1 -1066.3

Pareodiniaceratophora /dn -1322.2 -1322.2

Phoberocystaneocomica /dn -1340 -966.8

Polygonifera evittii /dn -1322.2 -1322.2

Pseudoceratiumpelliferum /dn -1283.5 -1113.9

Scriniodiniumattadalense /dn -1176.2 -950.0

Spiniferitesdentatus /dn -1066.3 -950.0

Spiniferites ramosus /dn -1192.0 -950.0

Subtilisphaeraperlucida /dn -1073.2 -950.0

Subtilisphaeratuerrula /dn -1080.9 -950.0

Systematophorafasciculigera /dn -1218.5 -1218.5

Tanyosphaeridium salpinx /dn -1302.5 -950.0

Tanyosphaeridiumvariecalamus /dn -1073.2 -950.0

Wallodiniumcylindrica /dn -1243.1 -950.0*=Prismatocystis

Wallodiniumkrutzschii /dn -1277.0 -950.0

LOK.2 DSDP 535 Southeastern Gulf of Mexico, 23° 42.48'N, 84° 30.97'W;

Buffler, R.T., et al., 1984, Initial Reports DSDP 77, Washington (U.S. Govt. Printing Office).

Cherchi& Schroeder, 1984, p. 585-587 - transported, reworked??

Dictyoconuswalnutensis /fb -202.2 -200.6

Paracoskinolinasunnilandensis /fb -202.0 -202.0*Id as cf.

Orbitolina texana /fb -202.2 -200.6

Premoli Silva & McNulty, 1984, p. 547-584

Calpionella alpina /ca -706.8 -684.0

Calpionellitesdarderi /ca -683.7 -641.0

Calpionellopsisoblonga /ca -706.8 -641.0

Calpionellopsis simplex /ca -714.0 -706.8

Lorenziella hungarica /ca -706.8 -684.0

Remaniellacadischiana /ca -692.8 -692.8

Remanielladadayi /ca -692.8 -692.8

Tintinnopsellacarpathica /ca -706.8 -641.0

Tintinnopsella longa /ca -706.8 -641.0

Premoli Silva & McNulty, 1984, p. 547-584

Globuligerinahoterivica /fp -570.6 -429.9

Gubkinellagraysonensis /fp -524.9 -524.9

Watkins &Bowdler, 1984, p. 649-674

Axopodorhabdusdietzmannii /nn -674.9 -443.6

Discorhabdus ignotus /nn -674.9 -437.0

Biscutumellipticum /nn -437.0 -437.0

Braarudosphaeraregularis /nn -474.7 -474.7

Calcicalathina oblongata /nn -648.2 -437.0 FO early Valanginian

Chiastozygusstriatus /nn -520.6 -443.6

Corollithionellipticum /nn -537.8 -537.8

Corollithion signum /nn -537.8 -537.8

Cretarhabdus conicus /nn -674.9 -437.0

Cretarhabdus loriei /nn -543.9 -437.0

Cribrosphaerellaehrenbergii /nn -537.8 -537.8

Cruciellipsiscuvillieri /nn -707.6 -443.6

Cyclagelosphaeradeflandrei /nn -707.6 -437.0

Cyclagelosphaeramargerelii /nn -707.6 -437.0

Diadorhombus rectus /nn -613.2 -575.5

Diazomatolithuslehmanii /nn -707.6 -437.0

Discorhabdusbiradiatus /nn -458.8 -451.7

Ethmorhabdusgallicus /nn -707.6 -466.7

Grantarhabdusmeddii /nn -530.6 -530.6

Haqiuscircumradiatus /nn -611.3 -437.0

Hayesitesatlanticus /nn -520.6 -451.7

Hayesites radiatus /nn -466.7 -437.0

Heleneachiastius /nn -674.9 -437.0

Lithraphiditesbollii /nn -466.7 -437.0

Lithraphiditescarniolensis /nn -707.6 -437.0

Manivitellapemmatoidea /nn -648.2 -437.0

Micrantholithushoschulzii /nn -674.9 -437.0

Micrantholithusobtusus /nn -707.6 -437.0

Nannoconus bermudezii /nn -565.5 -437.0

Nannoconusbroennimannii /nn -707.6 -437.0

Nannoconus colomii /nn -707.6 -437.0

Nannoconus cornutus /nn -693.7 -437.0

Nannoconuskamptneri /nn -512.3 -437.0

Nannoconussteinmannii /nn -707.6 -437.0

Parhabdolithusinfinitus /nn -613.2 -458.8

Reinhardites elegans /nn -520.6 -443.6

Reinharditesfenestratus /nn -674.9 -437.0

Retecapsaangustiforata /nn -707.6 -437.0

Rhagodiscus angustus /nn -466.7 -466.7

Rhagodiscus asper /nn -707.6 -437.0

Rhagodiscusreightonensis /nn -530.6 -443.6

Rotelapilluslaffittei /nn -707.6 -437.0

Rucinolithus wisei /nn -707.6 -512.3

Speetonia colligata /nn -707.6 -466.7

Stradneria crenulata /nn -674.9 -437.0

Tranolithus salillium /nn -707.6 -474.7

Tubodiscusverenae /nn -658.0 -537.8

Vekshinella stradneri /nn -707.6 -437.0

Watznaueria barnesae /nn -707.6 -437.0

Watznaueria biporta /nn -693.7 -437.0

Watznaueriabritannica /nn -707.6 -443.6

Watznaueria communis /nn -707.6 -443.6

Zeugrhabdotusembergeri /nn -707.6 -437.0

Zygodiscus erectus /nn -707.6 -437.0

Riley & Fenton, 1984, p. 675-690

Achomosphaeraneptuni /dn -693.4 -437.4

Alvellodiniumfalsificum /dn -611.3 -592.5

Apteodinium maculatum /dn -581.4 -465.0

Batioladiniumgochtii /dn -611.3 -511.7

Batioladiniumvarigranosa /dn -527.6 -527.6*=Aprobolocysta

Caddasphaera halosa /dn -667.2 -509.5

Callaiosphaeridiumasymmetricum /dn -465.0 -465.0

Chlamydophorellahuguoniotii /dn -552.5 -459.6

Cometodinium? whitei /dn -696.4 -608.9

Ctenidodiniumscissum /dn -558.7 -558.7

Cymatiosphaeradelicatula /dn -482.6 -447.2

Dapsilidiniumwarrenii /dn -705.5 -437.4 *=Polysphaeridium

Dicanthumhollisteri /dn -696.4 -552.5

Dingodinium albertii /dn -635.1 -437.4

Discorsia nanna /dn -459.6 -459.6

Druggidiumapicopaucicum /dn -683.7 -683.7

Druggidium deflandrei /dn -516.5 -516.5

Druggidiumrhabdoreticulatum /dn -611.6 -487.4

Endoscrinium glabra /dn -593.2 -592.5*=Athigmatocysta

Endoscrinium campanula /dn -598.8 -458.3*=Scrinodinium

Fromea amphora /dn -520.8 -520.8

Gonyaulacystahelicoidea /dn -696.4 -429.2

Gonyaulacystakostromiensis /dn -592.5 -429.2

Hystrichodiniumfurcatum /dn -611.6 -429.2

Hystrichodiniumpulchrum /dn -705.5 -429.2

Hystrichodiniumvoigtii /dn -641.5 -516.5

Kleithriasphaeridiumcorrugatum /dn -635.1 -635.1

Kleithriasphaeridiumeoinodes /dn -458.3 -458.3

Kleithriasphaeridiumfasciatum /dn -632.9 -534.7

Kleithriasphaeridiumsimplicispinum /dn -627.8 -593.2

Muderongia extensiva /dn -571.8 -568.3

Muderongia perforata /dn -527.6 -479.9

Muderongia simplex /dn -696.4 -479.9

Occisucysta tentorium /dn -693.4 -552.5

Oligosphaeridium complex /dn -641.5 -429.2

Oligosphaeridiumpulcherrimum /dn -552.5 -437.7

Pareodiniaceratophora /dn -677.7 -527.6

Phoberocystaneocomica /dn -705.5 -437.4

Phoberocystatabulata /dn -656.4 -656.4

Pseudoceratiumpelliferum /dn -683.7 -458.3

Pterospermellaaureolata /dn -673.7 -429.2

Pterospermellaaustraliensis /dn -651.8 -641.5

Sirmiodinium grossii /dn -504.3 -504.3

Spiniferites ramosus /dn -641.5 -429.2

Tanyosphaeridium boletus /dn -705.5 -428.7

Trichodiniumcastanea /dn -598.8 -457.3

Wallodiniumcylindrica /dn -705.5 -527.6*=Prismatocystis

Wallodiniumkrutzschii /dn -705.5 -447.2

Wallodinium lunua /dn -476.1 -428.7

LOK.3 Rio Argos section, Caravaca, Spain, 38.085525, -1.89343;

Hoedemaeker&Leereveld, 1995, Cretaceous Research, 16:195-230; Coccioni&Premoli-Silva, 1994, Cretaceous

Research, 15:645-687.

Base Barremian /mb 789 * base S. oosteri

Base Hauterivian /mb 472 * base B. oosteri = radiatus zone

Base Late Valanginian /mb * *

Base Valanginian /mb 264 *= base T. pertransiens

Base Berriasian /mb 10 *= base B. jacobi

Clavihedbergellaeocretacea /fp 828 1132

Clavihedbergellasemielongata /fp828 1132

Glob'oides aptiense /fp 1030 1060

Glob'oides blowi /fp 980 1132

Glob'oides gottisi /fp 416 1132

Glob'oidesmaridalensis /fp 1132 1332

Globuligerinahoterivica /FP 311 1128 ID as Favusella

Gubkinellagraysonensis /fp 828 1128 very rare at 458m

Hedbaptiana /fp 828 1132

Hedbaptica /fp 311 1132

Hedbdelrioensis /fp 434 1132

Hedbexcelsa /fp 1128 1128

Hedbkuznetsovae /fp 828 1128

Hedb sigali /fp 311 1128

Hedbsimilis /fp 813 1132

Leupoldina cabri /fp 1132 1132

Leupoldina pustulans /fp 828 1132

Calpionellitesdarderi /ca 307 307

Calpionellopsisoblonga /ca 200 200

Calpionellopsis simplex /ca 155 155

Crassicollariaparvula /ca 81 81

Lorenziella hungarica /ca 225 225

Remaniellacadischiana /ca 28 28

Remaniella murgeanui /ca 300 300

Ammonites from Hoedemaeker&Leereveld, 1995, Cret. Res., 16:195-230

Ancylocerasvandenheckii /am 900 900

Avramidiscus kiliani /am 794 813

Balearites balearis /am 747 755

Barremites spp. /am 778 1114

Berriasellabebrovensis /am 128 228

Berriasella callisto /am 173 260

Berriasellachomeracensis /am 10 70

Berriasella jacobi /am 10 40

Berriasella malbosi /am 45 45

Berriasellaparamacillenta /am 32 68

Berriasella picteti /am 129 241

Berriasellaprivasensis /am 78 152

Berriasellasubcallisto /am 41 86

Bochianitesneocomiensis /am 330 440

Bochianites oosteri /am 472 474

*Busnardoitescampylotoxum /am 333 374

Cheloniceras spp. /am 1128 1128

Colchidites spp. /am 1030 1104

*Crioceratitesduvali /am 658 691 base too low

Crioceratites loryi /am 506 506

Cruasiceras cruasense /am 681 681

Dalmasiceras dalmasi /am 127 127

Dalmasiceraspunctatum /am 145 162

Deshayesites weissi /am 1104 1113

Elenicerastchechitevi /am 438 438

Erdenellapaquieri /am 202 295

Erdenella zianidia /am 166 196

Fauriella boissieri /am 172 264

Fauriella gallica /am 127 202

Fauriella kiliani /am 235 320

Fauriellararefurcata /am 190 241

Heinziaprovincialis /am 900 900

Heinziasartousi /AM 1014 1021*=H. sartousiana

Himantocerastrinodosum /am 424 438

Holcodiscuscaillaudianus /am 840 869

Kilianella busnardoi /am 158 166

Kilianellaretrocostata /am 235 342

Kilianella roubaudi /am 281 336

Mazenoticerasparamimounum /am 145 202

*Mazenoticerasis subjective synonym of Malbosiceras fide Wright 1996

Neocomitesneocomiensis /am 260 339

Neocomitessubquadratus /am 300 320

Olcostephanustenuituberculatus /am 589 589

Olcostephanusbalestrai /am 420 428

Olcostephanushispanicus /am 474 474

Olcostephanusjeannoti /am 504 575

Olcostephanusdensicostatus /am 474 474

Oosterella garciae /am 418 440

Plesiospitidiscussubdifficilis /am 759 773

Pseudosaynellatermieri /am 1128 1128

Pseudosubplaniteseuxinus /am 32 42

Pseudosubplaniteslorioli /am 30 70

Pseudosubplanitesponticus /am 18 54

Pseudothurmanniacatulloi /am 775 790

Pseudothurmanniaohmi /am 764 792

Sarasinella eucyrta /am 280 290

Sarasinellatrezanensis /am 292 311

Spitidiscus fasciger /am 587 657

Spitidiscushugii /am 792 810

*Spitidiscus rawsoni /am 473 475 base too low

Subpulchellianicklesi /am 809 833

Subsaynella sayni /am 680 691

Subthurmanniaclareti /am 120 120

Subthurmanniafloquinensis /am 67 83

Subthurmanniaoccitanica /am 155 185

Subthurmanniapatruliusi /am 122 153

Subthurmanniasubalpina /am 75 88

Taveridiscus oosteri /am 789 800

Teschenites flucticulus /am 473 474

Teschenites neocomiensiformis /am 418 474

Thurmannicerasotopeta /am 266 310

Tirnovellaalpillensis /am 227 271

Tirnovellapertransiens /am 264 336

Valanginitesbachelardi /am 360 360

Dinoflagellates from Hoedemaeker&Leereveld, 1995, Cret. Res., 16:195-230

Amphorula delicata /dn 236 288

Aprobolocystaeilema /dn 695 754

Biorbiferajohnewingii /dn 149 384

Cymososphaeridiumvalidum /dn 401 775

Dicanthumhollisteri /dn 149 432

Dingodinium europaeum /dn 908 1026

Druggidiumapicopaucicum /dn 319 695

Druggidium deflandrei /dn 432 1033

Druggidiumrhabdoreticulatum /dn 705 911

Foucheria modesta /dn 236 385

Meiourogonyaulaxpertusapertusa /dn 483 695

Meiourogonyaulaxstoveri /dn 623 1038

Muderongia staurota /dn 483 937

Odontochitinaoperculata /dn 917 1038

Pseudoceratiumanaphrissum /dn 917 1028

Pseudoceratiumpelliferum /dn 222 1026

Subtilisphaeraperlucida /dn 754 1038

Subtilisphaera senegalensis /dn 809 1038

Systematophorapalmula /dn 149 433

Tanyosphaeridiummagneticum /dn 236 1026

LOK.5 Santa Rosa Canyon, Highway 58 west of Linares, Nuevo Leon, Mexico, 24.743731. -99.775644;

Blauser& McNulty, 1980, Trans. Gulf Coast Assoc. Geol. Soc., 30:263-272; Ice & McNulty, 1980, idem, 30:403-425.

Base Aptian /mb 2560 *

Base Albian /mb 3140 *

Marker bed Al SB WA 1 /mb 3195 3195

Marker bed Ap SB PR 1 /mb 2500 2500

Calpionellaalpina /ca 20 49

Calpionella elliptica /ca 20 197 *FO at base Berriasian

Calpionellitesdarderi /ca 295 295

*ID as Calpionelladarderi in Valanginian

Calpionellopsisoblonga /ca 49 436

Calpionellopsis simplex /ca 118 197

Remaniellacadischiana /ca 39 49

Stenosemellopsishispanica /ca 295 295

Tintinnopsellacarpathica /ca 20 463

Tintinnopsella longa /ca 49 410 *FO in lower Berriasian

Colomiella mexicana /ca 2676 2808

Colomiella recta /ca 2676 3011

Pith ovalis /ca 3395 3395

Pith sphaerica /ca 3198 4023

Nannoconussteinmannii /nn 39 2258

Nannoconus wassallii /nn 2460 2558

Microcalamoidesdiversus /id 2676 3198

Bitibreggiensis /fp 3211 3415

Glob'oides algeriana /fp 2519 2538

Glob'oidesbentonensis /fp 3467 3742

Glob'oides cushmani /fp 3461 4129

Hedbergellawashitensis /fp 2676 3447

Helv'anahelvetica /fp 4211 4211

Marg schneegansi /fp 4244 4244

Planomalina buxtorfi /fp 3421 3493

Praeglobotruncanadelrioensis /fp 3447 3742

Praeglobotruncanastephani /fp 3447 4096

Rota appenninica /fp 3428 3742

Rota cushmani /fp 4031 4129

Rota gandolfi /fp 3900 3900

Rota greenhornensis /fp 3949 3949

Ticiroberti /fp 3198 3480

Ticisubticinensis /fp 3224 3410

Ticiticinensis /fp 3326 3493

LOK.6 Berrias Section, France, 44.388252, 4.202586;

**Galbrun**et al., 1986, Donnesnouvelles sur le stratotype du Berriasien: correlations entramagnetostratigraphie et biostratigraphie. Bulletin Societe geologic France, 8:575-584, figs. 2, 6.

Berriasella callisto /am 24 25.5

Berriasellaparamacillenta /am 1 7

Berriasella picteti /am 22 22.5

Berriasellaprivasensis /am 8 10

Berriasellasubcallisto /am 7 7

Dalmasiceras dalmasi /am 11 11

Dalmasiceraspunctatum /am 11 11

Erdenella paquieri /am 22 22

Fauriellaboissieri /am 22 22

Holcophylloceras calypso /am 1 22.5

Mazenoticerasparamimounum /am 13.5 15

*Mazenoticeras a subjective synonym of Malbosiceras fide Wright 1996

Ptychophyllocerassemisulcatum /am 7 10

Subthurmanniaoccitanica /am 10 10

Subthurmanniasubalpina /am 7 8

Conusphaera mexicana /nn 0 3

Cretarhabduscrenulatus /nn 14 18

Cruciellipsiscuvillieri /nn 14 18

Cyclagelosphaeramargerelii /nn 0 3

Diazomatolithuslehmanii /nn 0 22

Lithraphiditescarniolensis /nn 5 5

Micrantholithusobtusus /nn 18 22

Miculainfracretacea /nn 18 22

Nannoconusbroennimannii /nn 14 16

Nannoconus colomii /nn 5 22

Nannoconus globulus /nn 14 16

Parhabdolithusembergeri /nn 5 22

Watznaueria barnesae /nn 0.5 13

Watznaueriabritannica /nn 5 22

Watznaueria communis /nn 0 22.5

LOK.6B Berrias Section, France, 44.388252, 4.202586;

**Le Hegarat**, 1968, Tithoniquesuperieur et Berriasien de LeArdeche et de LeHerault correlation des ammonites et des calpionelles. Geobios 1:7-70, Tab. V; Le Hegarat, G., 1971, De Berriasian du sud-est de la France. Document LaboratoireGeologie Faculty‚ Sciences de Lyon 43:1-308, Tab. 17.

Berriasellacallisto /am 22.5 23.5

Berriasellaparamacillenta /am 5 8

Berriasella picteti /am 20 21

Berriasellaprivasensis /am 9 11

Berriasellasubcallisto /am 8 8

Dalmasicerasdalmasi /am 12 12

Dalmasiceraspunctatum /am 12 12

Erdenella paquieri /am 20 20 as Jabronella

Mazenoticerasparamimounum /am 14 14

Fauriellararefurcata /am 20 21

Kilianellapexiptycha /am 24.5 26.5

Neocomitesneocomiensis /am 25.5 28

Subthurmanniaoccitanica /am 11 11 as Tirnovella

Subthurmanniasubalpina /am 8 9

Thurmannicerasgratianopolitense /am 24.5 24.5

Thurmanniceraspertransiens /am 26.5 28

Calpionella alpina /ca 3 24

Calpionella elliptica /ca 11.5 14

Calpionellitesdarderi /ca 24.5 24.5

Calpionellopsisoblonga /ca 17 24

Calpionellopsis simplex /ca 14.2 23

Crassicollariaparvula /ca 3 12.5

Lorenziella hungarica /ca 23 28

Lorenziella plicata /ca 22 23

Remaniellacadischiana /ca 8 28

Tintinnopsellacarpathica /ca 3 28

Tintinnopsella longa /ca 17 26

LOK.7 ODP 638B & C, Offshore Spain; 42° 09.2N, 12° 11.8W;

Applegate & Bergen, 1988, Proc. ODP, Sci. Results, v. 103, p. 293 ff; Valanginian-Aptian, Table 2.

Assipetrainfracretacea /NN -463.3 -184.3

Axopodorhabdusdietzmannii /nn -499.2 -184.3

Biscutum constans /nn -499.2 -184.3

Bukrylithus ambiguus /nn -499.2 -191.1

Calcicalathina oblongata /nn -473.2 -212.5

Chiastozyguslitterarius /nn -219.8 -191.11st consistent base, lowest base @ 306.3

Chiastozygusplatyrhethus /nn -217.3 -192.1

Conusphaera mexicana /nn -499.2 -184.3

Corollithion acutum /NN -384.1 -197.0

Corollithionellipticum /NN -499.2 -184.3

Cretarhabdus conicus /nn -499.2 -184.3

Cretarhabdusloriei /nn -326.4 -192.1 ID as cf.

Cretarhabdussurirellus /nn -499.2 -184.3

Cruciellipsiscuvillieri /nn -499.2 -193.7

Cyclagelosphaeramargerelii /nn -499.2 -184.3

Diadorhombus rectus /nn -499.2 -192.1

Diazomatolithuslehmanii /nn -499.2 -184.3

Discorhabdus ignotus /nn -499.2 -184.3

Flabellites oblonga /nn -221.9 -200.1

Grantarhabdusmeddii /nn -499.2 -184.3

Haqiuscircumradiatus /nn -499.2 -184.3

Lithraphiditescarniolensis /nn -499.2 -184.3

Manivitellapemmatoidea /nn -499.2 -184.3

Micrantholithushoschulzii /nn -499.2 -184.3

Micrantholithusobtusus /nn -499.2 -184.3

Nannoconus bermudezii /NN -499.2 -192.1

Nannoconus bucheri /NN -329.7 -191.1

Nannoconus elongatus /NN -329.7 -216.2

Nannoconus globulus /NN -499.2 -191.1

Nannoconus kamptneri /NN -329.7 -200.1

Nannoconussteinmannii /NN -499.2 -184.3

Nannoconus truitti /nn -329.7 -184.3

Nannoconus wassallii /NN -211.9 -192.1

*lowest base @ 306.3 followed by long gap

Parhabdolithusjudithae /nn -490.1 -415.0

Percivalia fenestrata /nn -499.2 -184.3

Pickelhaube furtiva /nn -499.2 -248.42nd top,1st @ 211.9

Rhagodiscus asper /NN -499.2 -184.3

Rhagodiscus splendens /NN -499.2 -184.3

Rucinolithusterebrodentarius /nn -200.1 -191.1

Rucinolithus wisei /nn -424.8 -384.4

Sollasites horticus /nn -499.2 -184.3

Speetonia colligata /nn -499.2 -213.4

Stephanolithionlaffittei /nn -499.2 -184.3

Tegumentum stradneri /nn -344.7 -184.3

Tetrapodorhabduscoptensis /NN -499.2 -184.3

Tetrapodorhabdusdecorus /NN -499.2 -306.3

Tranolithus gabalus /nn -499.2 -184.3

Tubodiscusjurapelagicus /nn -499.2 -184.3

Tubodiscus verenae /nn -499.2 -220.9

Watznaueriabarnesae /nn -499.2 -184.3

Watznaueria biporta /nn -499.2 -184.3

Watznaueriabritannica /nn -499.2 -229.3

Zeugrhabdotusembergeri /NN -499.2 -184.3

Zeugrhabdotuspseudoangustus /NN -499.2 -192.1

Masure, 1988, Figs. 2, 3, p. 435 & 437

Achomosphaeraneptuni /dn -538.8 -307.7

Batioladiniumvarigranosa /dn -434.6 * =Aprobolocysta

Biorbiferajohnewingii /dn -471.8 -317.8

Bourkidiniumgranulatum /dn -214.2 *

Canninginopsiscolliveri /DN -245.0 -211.1

Cassiculosphaeridiareticulata /dn -252.0 -191.3 ID as cf.

Cerbiatabulata /dn -211.1 -191.3

Chlamydophorellanyei /dn -307.7 -270.3

Cometodinium? whitei /dn -344.7 -211.1

Ctenidodiniumelegantulum /dn -317.8 -278.1

Cyclonepheliumdistinctum /DN -266.1 -211.1

Cyclonepheliumhystrix /DN -538.8 -225.5

Dapsilidiniumwarrenii /dn -528.1 -192.8

Dingodiniumcerviculum /DN -471.8 -192.8

Druggidiumapicopaucicum /dn -471.8 -214.2

*base in Valanginian

Druggidium deflandrei /dn -252.0 -191.3

Druggidiumrhabdoreticulatum /dn -252.0 -191.3

Exiguisphaera phragma /dn -434.6 -307.7

Heterosphaeridium? galliciae /dn -344.7 -211.1

Hystrichodiniumfurcatum /dn -307.7 *

Hystrichodiniumpulchrum /dn -191.3 -191.3

Hystrichodiniumvoigtii /dn -462.4 *

Kiokansium polypes /dn -434.6 -211.1

Kleithriasphaeridiumeoinodes /dn -211.1 -192.8

Kleithriasphaeridiumfasciatum /dn -307.7 -192.8

Meiourogonyaulaxpertusapertusa /dn -402.5 *

Meiourogonyaulaxstoveri /dn -214.2 -192.8

Muderongia perforata /dn -307.7 -245.0

Muderongia simplex /dn -538.8 -501.3

Muderongia simplex microperforata /dn -434.6 *

Oligosphaeridium complex /dn -245.0 -225.5

Oligosphaeridiumdividuum /dn -278.1 -214.2

Oligosphaeridiumpulcherrimum /dn -225.5 -214.2

Oligosphaeridiumverrucosum /dn -192.8 *

Phoberocystaneocomica /dn -538.8 -211.1

Protoellipsodiniumseghire /dn -245.0 -225.5

Protoellipsodiniumtouile /dn -270.3 -214.2

*subsp. mugatae; base in Hauterivian

Pseudoceratiumpelliferum /dn -538.8 -325.7

Rhynchodiniopsisaptiana /dn -225.5 -192.8

Rhynchodiniopsis fimbriata /dn -434.6 *

Spin ramosusmultibrevis /DN -462.4 -214.2

Spiniferites ramosus /dn -402.5 -214.2

Systematophoraareolata /dn -538.8 -431.8

Systematophorasilybum /dn -402.5 -214.2

Tanyosphaeridiummagneticum /dn -480.3 -431.8

Wallodiniumkrutzschii /dn -266.1 -192.8

LOK.8 Bosso Valley, Italy, 43° 31.17, 12° 34.27E;

Housa et al., 2004, Combined magnetostratigraphic, paleomagnetic and calpionellid investigations across Jurassic/Cretaceous boundary strata in the Bosso Valley, Umbria, central Italy. Cretaceous Research 25:771-785; Jur/Cret boundary section. **Magnetochrons.**

Calpionella alpina /ca 6 29.6

Calpionellagrandalpina /ca 6 14

Chitinoidellaslovenica /ca -5.8 3.6

Chitinoidellaboneti /ca 4.6 4.6

Crassicollariabrevis /ca 6.8 12.5

Crassicollaria intermedia /CA 7 12.4

Crassicollariamassutiniana /CA 6.2 13.2

Crassicollariaparvula /CA 6 15.6

Praetintinnopsellaandrusovi /ca 3.8 4.6

Tintinnopsellacarpathica /ca 5.2 29.6

Magnetochron M17r /MA * 30 Top ofsection @ 30m

Magnetochron M18n /MA * 28.5

Magnetochron M18r /ma * 23.5

Magnetochron M19n /MA * 21.1

Magnetochron M19n.1r /ma * 19.6

Magnetochron M19r /ma * 8.0

Magnetochron M20n /MA * 6.2

Magnetochron M20n.1r /ma * -0.4

*Lowest sample @ -9 m, may not be accurate base M20n

LOK.10 Barret-le-Bas Section, France, 44.26213, 5.73260;

Busnardo et al. 1979, p. 44, fig. 13; p. 90, fig. 28; p. 105, fig. 30; Bulot, 1995, Ph.D., figs. 3,6, 7.

Baronnites hirsutus /am 62 72

Berriasella callisto /am 0 1

Bochianitesneocomiensis /am 57 105

Busnardoitescampylotoxum /am 98 110

Busnardoites desori /am 81 110 ID as cf.

Busnardoitessubcampylotoxum /am 75 81

Clavithurmanniaforaticostata /am 0 5.5

Erdenellapaquieri /AM 0 3

Fauriella boissieri /am 0 4.5

Fauriella donzei /am 0 3

Fauriella kiliani /am 2 26

Holcophylloceras calypso /am 36 71

Kilianellalucensis /am 29 96

Kilianella pexiptycha /am 1.5 9.5

Kilianellaretrocostata /am 5.5 57

Kilianella roubaudi /am 62 96

Luppovella superba /am 66 75

Lytocerasjuileti /am 68 92

Neolissocerasgrasianum /am 57 96

Neocomitesneocomiensis /am 103 119

Neocomites premolicus /am 26 57

Neocomites subtenuis /am 113 119

Neocomitesteschenensis /am 71 84

Neolisocerassalinarium /am 54 102

Olcostephanusdrumensis /am 26 65

Olcostephanusguebhardi /am 98 102

Olcostephanusjosephinus /am 62 72

Olcostephanusstephanophorous /am 62 108

Olcostephanustenuituberculatus /am 68 110

Phylloceras tethys /am 60 72

Platylenticerascardioceroides /am 62 65

Platylenticerasoccidentale /am 46 68

Protancyloceraspunicum /am 0 2

Protetragonitesquadrisulcatus /am 26 92

Ptychophyllocerassemisulcatum /am 31 96

Saynoceras verrucosum /am 119 119

Thurmannicerasotopeta /am 1 9

Thurmanniceraspertransiens /am 26 52

Tirnovellaalpillensis /am 0 9.5

Valanginitesbachelardi /am 119 119

Biscutumconstans /nn 0 125

Braarudosphaerabigelowii /nn 0 125

Calcicalathinaoblongata /nn 46 125

Conusphaera mexicana /nn 0 125

Cretarhabdus conicus /nn 0 125

Cretarhabduscrenulatus /nn 0 125

Cretarhabdussurirellus /nn 1 125

Cruciellipsischiasta /nn 0 125

Cruciellipsiscuvillieri /nn 0 125

Cyclagelosphaeramargerelii /nn 0 125

Diadorhombus rectus /nn 67 125

Diazomatolithuslehmanii /nn 0 125

Discorhabdusrotatorius /nn 0 125

Lithraphiditescarniolensis /nn 0 125

Manivitellapemmatoidea /nn 0 125

Markaliuscircumradiatus /nn 0 125

Micrantholithushoschulzii /nn 0 125

Micrantholithusobtusus /nn 1 125

Miculainfracretacea /nn 50 125

Nannoconusbroennimannii /nn 4 97

Nannoconus colomii /nn 0 125

Nannoconus globulus /nn 26 120

Parhabdolithus asper /nn 0 125

Parhabdolithusembergeri /nn 0 125

Parhabdolithussplendens /nn 46 125

Podorhabdusdietzmanni /nn 46 97

Reinharditesfenestratus /nn 41 125

Rhabdolithus rectus /nn 7 125

Rucinolithus wisei /nn 10 50

Speetonia colligata /nn 41 125

Stephanolithionlaffittei /nn 0 125

Tubodiscus verenae /nn 2 78

Vagalapilla compacta /nn 0 92

Vagalapilla stradneri /nn 0 125

Watznaueria barnesae /nn 0 125

Watznaueria biporta /nn 0 92

Watznaueriabritannica /nn 0 125

Watznaueria communis /nn 0 125

Zygodiscus elegans /nn 1 125

Zygodiscusdiplogrammus /nn 97 125

Calpionella alpina /ca 0 12

Calpionellitescoronata /ca 71 87

Calpionellitesdarderi /ca 28 87

Calpionellopsis oblonga /ca 0 36

Lorenziellahungarica /ca 0 70

Remaniellacadischiana /ca 1 35

Remaniella murgeanui /ca 28 32

Tintinnopsellacarpathica /ca 0 87

Tintinnopsella longa /ca 3 87

LOK.11 Angles Section, France, 43.82992, 6.22561;

Busnardo, R., Thieuloy, J.-P., Moullade, M., 1979, Hypostratotype Mesogéen de l’etageValanginien (sud-est de la France). Les StratotypesFrançais, v. 6, Centre National de la Recherche Scientitque.

Marker bed 305 /mb 111 *

Marker bed 321 /mb 125 *

Acanthodiscus radiatus /am 226 227

Acanthodiscusrebouli /am 226 227

Bochianitesneocomiensis /am 54 227

Bochianites oosteri /am 226 230

Breistrofferella castellanensis /am 239 243

Breistrofferellavarappensis /am 227 241

Busnardoitescampylotoxum /am 67 88

Clavithurmanniaforaticostata /am 6 9

Criosarasinellafurcillata /am 195 200

Eleniceras nikolovi /am 222 239

Elenicerastchechitevi /am 195 227

Dichotomites vergunnorum /am 185 191

Fauriella boissieri /am 2 9

Fauriella kiliani /am 18 20

Himantocerastrinodosum /am 179 188

Hypophyllocerascourchonense /am 178 191

Hypophyllocerasperlobatum /am 97 119

Kilianella lucensis /am 43 45

Lytoceras juileti /am 97 133

Neocomitesneocomiensis /am 97 185

Neocomitessubquadratus /am 99 110

Neocomites subtenuis /am 111 133

Neocomitesteschenensis /am 111 123

Neohoplocerasprovinciale /am 111 133 ID as gr.

Neohoplocerassubmartini /am 111 128

Neolissocerasaberrans /am 110 110

Neolissocerasgrasianum /am 54 245

Olcostephanus densicostatus /am 187 238

Olcostephanus drumensis /am 99 109

Olcostephanusnicklesi /am 185 191

Olcostephanusstephanophorous /am 99 109

Olcostephanustenuituberculatus /am 67 127

Oosterellacultrataeformis /am 235 241

Protetragonitesquadrisulcatus /am 91 191

Phylloceras tethys /am 91 210

Phyllopachyceraswinckleri /am 225 226

Ptychophyllocerasdiphyllum /am 111 191

Ptychophyllocerassemisulcatum /am 54 188

Sarasinella biformis /am 111 125

Sarasinella eucyrta /am 106 109

Sarasinellatrezanensis /am 100 109

Saynoceras verrucosum /am 111 123

Substrebliteszonarius /am 111 123

Teschenites callidiscus /am 211 215

Teschenites flucticulus /am 222 234

Teschenites pachydicranus /am 185 245

Thurmannicerasotopeta /am 8 11

Thurmannicerasperisphinctoides /am 11 15 ID as aff.

Thurmannicerassalientum /am 10 11 ID as cf

Valanginitesbachelardi /am 75 133 ID as cf.

Valanginites nucleus /am 111 123

Biscutum constans /nn 7 243

Braarudosphaerabigelowii /nn 7 243

Calcicalathinaoblongata /nn 65 243

Conusphaera mexicana /nn 5 243

Cretarhabdus conicus /nn 7 227

Cretarhabduscrenulatus /nn 5 243

Cretarhabdussurirellus /nn 5 243

Cruciellipsischiasta /nn 7 233

Cruciellipsiscuvillieri /nn 5 243

Cyclagelosphaeramargerelii /nn 5 243

Diadorhombus rectus /nn 120 150

Diazomatolithuslehmanii /nn 5 243

Discorhabdusrotatorius /nn 5 243

Lithraphiditescarniolensis /nn 5 243

Manivitellapemmatoidea /nn 7 243

Markaliuscircumradiatus /nn 7 243

Micrantholithushoschulzii /nn 5 243

Micrantholithusobtusus /nn 5 243

Miculainfracretacea /nn 130 243

Nannoconus colomii /nn 5 243

Nannoconus globulus /nn 21 196

Parhabdolithus asper /nn 7 243

Parhabdolithusembergeri /nn 5 243

Parhabdolithussplendens /nn 130 243

Podorhabdusdietzmanni /nn 95 227

Reinharditesfenestratus /nn 21 240

Rhabdolithus rectus /nn 56 168

Rucinolithus wisei /nn 28 56

Speetonia colligata /nn 60 243

Stephanolithionlaffittei /nn 5 243

Tubodiscus verenae /nn 7 212

Vagalapilla compacta /nn 7 206

Vagalapilla stradneri /nn 5 243

Watznaueria barnesae /nn 5 243

Watznaueria biporta /nn 7 219

Zygodiscus elegans /nn 7 240

Zygodiscusdiplogrammus /nn 95 240

Calpionella alpina /ca 0 36 ID as aff.

Calpionellitescaravacaensis /ca 47 64

Calpionellitescoronata /ca 36 64

Calpionellitesdarderi /ca 30 68

Calpionellopsis oblonga /ca 0 33

Calpionellopsis simplex /ca 0 11

Lorenziella hungarica /ca 0 44

Lorenziella plicata /ca 9 11

Remaniellacadischiana /ca 5 21

Remaniella murgeanui /ca 15 73

Tintinnopsellacarpathica /ca 0 64

Tintinnopsella longa /ca 0 60

*Marker bed (/mb) sets of more resistent limestone-marl bed sets;Bulot et al., 1992, 1995 Ph.D.Cycle F2 beds 233-243; Cycle F1 beds 224-base 232; Cycle N beds 218-219;

Cycle Vbeds 206-base 213; Cycle P beds 204-206 base.

LOK.12 Angles Section, Les Baoussayes, Vocontian Basin, France, 43.82992, 6.22561;

Bulot et al. 1992, 1995, spans upper Valanginian-lower Hauteriviancampylotoxus - radiatus zones.

Marker bed 305 /mb 6 *

Marker bed 321 /mb 23 *

Base Upper Valanginian /mb 6 *

*proposed GSSP @ base of Saynocerasverrucosum

Acanthodiscus radiatus /am 96 107

Breistrofferella castellanensis /am 96 105

Criosarasinellafurcillata /am 76 84

Criosarasinellamandovi /am 81 87

Eleniceras nikolovi /am 96.5 96.5

Himantocerastrinodosum /am 56 85

Jeanthieuloyitesquinquestriatus/am 67 73

Karakaschiceraspronecostatum /am 19 25

Neocomitesneocomiensis /am 0 20.5

Neocomites peregrinus /am 27 47 ID as Varlheideites

Neocomitesplatycostatus /am 6 7

Neocomitessubquadratus /am 0 5

Neocomites subtenuis /am 6 26

Neocomitesteschenensis /am 6 12

Neohoplocerasdepereti /am 19.5 26

Neohoplocerasprovinciale /am 1.5 10

Neohoplocerassubmartini /am 6 12

Olcostephanusbalestrai /am 37 68

Olcostephanus nicklesi /am 56 63

Olcostephanusstephanophorous /am 0 4

Olcostephanustenuituberculatus /am 0 10

Olcostephanusthieuloyi /am 70 75

Oosterellacultrata /am 90.5 90.5

Oosterella fascigera /am 32 51

Sarasinella biformis /am 6 10

Saynoceras verrucosum /am 6 10

Sarasinella hirticula /am 56 56

Teschenites callidiscus /am 87 93

Teschenites flucticulus /am 95.5 115

Teschenites pachydicranus /am 73 115

Teschenites subflucticulus /am 84 95.5

Valanginitesbachelardi /am 6 26

Valanginites nucleus /am 6 10

*Marker bed (/mb) sets of more resistent limestone-marl bed sets;

*Bulot et al., 1992, 1995 Ph.D. Cycle F2 beds 233-243; Cycle F1 beds 224-base 232; Cycle N beds 218-219; Cycle V beds 206-base 213; Cycle P beds 204-206 base.

LOK.13 LaCharce Section, Vocontian Basin, France, 44.53208, 5.3646; 1992;

Bulot, L., Thieuloy, J.-P., Blanc, E., 1992, Le cadre stratigraphique du Valanginiensupérior et de l’Hauterivien du Sud-Est de la France. Geologie Alpine 68:13-56; Tables VI, p. 27, VII, p. 30, VIII, p. 35, XII, p. 44, XIV, p. 49. Section spans Valanginian/Hauterivian boundary to upper Hauterivian Stage.

*Data in fig. 5.7 and table in Bulot's dissertation 1995.

Carbon peak IntraVal OAEb /gc -2.0 *** (Lower Hauterivian)

Carbon peak IntraValOAEc /gc -25 ***

Carbon peak IntraValOAEd /gc -118 -120

These three peaks form the “Weissert Anoxic Event”

Base Hauterivian /mb -93.5 ***

At base A. radiatus/base Radiatus Zone; but see LOK.13b at base A. rebouli.

Acanthodiscus radiatus /am -93.5 -107

Acanthodiscusrebouli /am -91 -108

Breistrofferellacastellanensis /am -96 -102

*Base Castellanensis Subzone of Radiatus Zone

Crioceratitesduvalii /am -191 -193

Crioceratites gr. quenstedti /am -149 -171

Crioceratites loryi /am -119 -130

*Base Loryi Subzone/Zone

Crioceratitesmatsumotoi /am -179 -184

Criohimantoceras gigas /am -39.5 -83

Criosarasinellafurcillata /am -64 -75

*Base FurcillataSubzoneofTrinodosumZone

Criosarasinellaheterocostata /am -64 -74

Criosarasinellamandovi /am -72 -74

Cruasiceras cruasense /am -169 -172

*Base C. cruasense Subzone

Dichotomites petschi /am -28 -43

Eleniceras nikolovi /am -93.5 -93.5

Eleniceras transsylvanicum /am -86 -92

Euptychoceras meyrati /am -149 -171

Himantoceras trinodosum /am -39.5 -95

Jeanthieuloyitesquinquestriatus/am -65 -92

Karakaschiceras pronecostatum /am -10 -25.5

*Base Pronecostatum Subzone of Verrucosum Zone

Leopoldia leopoldina /am -102 -107

Lyticoceras nodosoplicatum /am -143 -150 Base Nodosoplicatum Zone

Neocomites callidiscus /am -83 -86

Neocomitesflucticulus /am -90 -107

Neocomitesneocomiensis /am -1 -14

Neocomitesneocomiensiformis /am -1 -14

Neocomitespachydicranus /am -64 -107

Neocomites peregrinus /am -24 -43 ID as Varlheideites

Neocomitesplatycostatus /am -1 -1

Neocomites polygonius /am -78 -78

Neocomites subtenuis /am -1 -14

Neocomitesteschenensis /am -47 -47

Neohoplocerasdepereti /am -10 -34

Neohoplocerassubmartini /am -1 -10

Olcostephanusbalestrai /am -33 -73

Olcostephanusdensicostatus /am -65 -107

Olcostephanusjeannoti /am -130 -143 Base Jeannoti Subzone/Loryi Zone

Olcostephanusnicklesi /am -39.5 -54 Base Nicklesi Subzone/Trinodosum

Olcostephanus sayni /am -127 -133

Olcostephanustenuituberculatus /am -5 -14

Olcostephanus variegatus /am -142 -147 Base O. variegatus Subzone

Oosterella cultrata /am -69 -74

Oosterellacultrataeformis /am -61 -85

Oosterella fascigera /am -23 -47

Oosterellagarciae /am -83 -83

Oosterella stevenini /am -46 -69

Plesiospitidiscusligatus /am -203 -209 Base Ligatus Zone

Protacrioceraspuzosianum /am -143 -144

Rodighieroitesrutimeyeri /am -24 -34 ID as aff. or group

Sarasinella hirticula /am -34 -47

Saynellaclypeiformis /am -145 -147

Spitidiscus fasciger /am -133 -145

Spitidiscus gr. pavlowi /am -126 -145

Suboosterellaheliaca /am -137 -137

Subsaynella sayni /am -168 -191

*Base Sayni Zone = Base Upper Hauterivian

Teschenites callidiscus /am -83 -86

*Base Callidiscus Subzone of Callidiscus Zone

Teschenites castellanensiformis /am -96 -102

Teschenites flucticulus /am -90 -107

Varlheideitesperegrinus /am -24 -43

*Base Peregrinus Subzone of Verrucosum Zone

*Marker bed sets of more resistent limestone-marl bed sets;

*Bulot et al., 1992, 1995 Ph.D. Not included in composite dataset.

Cycle F2 beds 233-243; Cycle F1 beds 224-base 232; Cycle N beds 218-219;

Cycle V beds 206-base 213; Cycle P beds 204-206 base.

LOK.13bLa Charce Section, Vocontian Basin, France, 44.53208, 5.3646; 2008;

La Charce village 75 km NE of Avignon, SE France. Complements LOK.13 with new data. Reboulet, Chapter 2. Carnet de Geologie CG2008_BOOK_01, E. Mattioli, ed., fig. 2.1, p. 8 and fig. 3.1, p. 10. Section spans Valanginian/Hauterivian boundary to upper Hauterivian Stage.

*Ammonite Data from Fig. 2.1, p. 8

*Base Hauterivian = base A. radiatus Zone bed 189 (= Bulot bed 250) at FO A. rebouli

Acanthodiscus radiatus /am -8.0 -29.5

Acanthodiscusrebouli /am -8.0 -29.5 *In text p. 10

*Base A. radiatus Zone Bed 189/250

Breistrofferella castellanensis /am -9.0 -31.75ID as Breistrofferella

Himantocerastrinodosum /am 0 -0.25

Jeanthieuloyitesquinquestriatus/am 0 -10.25

Olcostephanusdensicostatus /am 0 -31.75

Olcostephanus sayni /am -35.75 -38.5

Oosterellacultrata /am 0 -2.0

Oosterellacultrataeformis /am 0 -2.0

Spitidiscus gr. lorioli /am -12.75 -31.75

Teschenites callidiscus /am 0 -5.25

Teschenites castellanensiformis /am -6.75 -13.75

Teschenites flucticulus /am -8.0 -30.75

Teschenites pachydicranus /am -8.0 -30.75

Teschenites subflucticulus /am 0 -2.0

Teschenites subpachydicranus /am 0 -7.0

*Gardin, Chapter 3. Nannofossils Fig. 3.1, p. 11

Amphizygusinfracretacea /nn 1.25 -52

Braarudosphaeradiscula /nn 2.25 -52

Calcicalathinaoblongata /nn 2.25 -52

Chiastozygus tenuis /nn 1.25 -52

Conusphaera mexicana /nn 2.25 -52

Cretarhabdus conicus /nn 2.25 -52

Cretarhabdussurirellus /nn 2.25 -52

Crucibiscutumsalebrosum /nn 2.25 -52

Cruciellipsiscuvillieri /nn 2.25 -52*LO = base Zone CC5

Cyclagelosphaeramargerelii /nn 2.25 -52

Diadorhombus rectus /nn 2.25 -52

Diazomatolithuslehmanii /nn 2.25 -52

Discorhabdusrotatorius /nn 2.25 -52

Eiffellithuswindi /nn 2.25 -30.75

*"best approximates Valanginian/Hauterivian boundary is the LO..."

Ethmorhabdusgallicus /nn 2.25 -52

Haqiuscircumradiatus /nn 2.25 -52

Lithraphiditesbollii /NN -51 -52

*FO = base Biozone CC4a w/ Eiffellithusstriatus

Lithraphiditescarniolensis /nn 2.25 -52

Manivitellapemmatoidea /nn 2.25 -52

Micrantholithushoschulzii /nn 1.25 -52

Micrantholithusobtusus /nn 2.25 -52

Microstauruschiastius /nn 2.25 -52

Microstaurus quadratus /nn 2.25 -52

Nannoconus bucheri /nn 2.25 -52

Nannoconus globulus /nn 0.75 -20.5

Nannoconus kamptneri /nn 2.25 -52

Nannoconus steinmannii /nn 2.25 -52

Nannoconus truitti /nn 1.25 -49

Nannoconus wassallii /nn -0.25 -52

Parhabdolithusinfinitus /nn 0.75 -49

Percivalia fenestrata /nn 1.25 -52

Pickelhaube furtiva /nn 0.75 -52

Retecapsaangustiforata /nn 2.25 -52

Rhagodiscus asper /nn 2.25 -52

Rotelapilluslaffittei /nn 2.25 -52

Sollasites horticus /nn 2.25 -52

Speetoniacolligata /nn 2.25 -52

Staurolithites crux /nn 2.25 -52

Tegumentum stradneri /nn -4.75 -52

Tubodiscusjurapelagicus /nn 0.75 -44

Tubodiscus verenae /nn 0.75 -50 LO = base Zone CC4

Watznaueria barnesae /nn 2.25 -52

Zeugrhabdotusembergeri /nn 2.25 -52

Zeugrhabdotuspseudoangustus /nn 0.75 -51

Zeugrhabdotustrivectis /nn 2.25 -52

Zygodiscusbicrescenticus /nn 1.25 -47

Zygodiscusdiplogrammus /nn 2.25 -52

LOK.14 Curnier Section, France, 44.38623, 5.23692;

Bulot, L., Thieuloy, J.-P., Blanc, E., 1992, Le cadre stratigraphique du Valanginiensupérior et de l’Hauterivien du Sud-Est de la France. Geologie Alpine 68:13-56; p. 22, Table III, Section spans upper Valanginian in part.

Himantocerastrinodosum /am 38 38

Karakaschiceraspronecostatum /am 29 29

Neocomites peregrinus /am 29.5 31 *ID as Varlheideites

Neocomites subtenuis /am 2 14

Neocomitesteschenensis /am 2 14

Neohoplocerassubmartini /am 9 9

Olcostephanus nicklesi /am 38 38

Saynoceras verrucosum /am 7 9

Valanginitesbachelardi /am 7 14

LOK.15 Moriez (St-Firmin) Section, France, 43.96183, 6.46985;

Bulot, L., Thieuloy, J.-P., Blanc, E., 1992, Le cadre stratigraphique du Valanginiensupérior et de l’Hauterivien du Sud-Est de la France. Geologie Alpine 68:13-56; p. 24, Table IV, Section spans lower-upper Valanginian in part.

Neocomitesneocomiensis /am 0.5 14

Neocomitessubquadratus /am 0.5 10

Neocomitessubtenuis /am 6 14

Neohoplocerasprovinciale /am 4 10

Neohoplocerassubmartini /am 9 12

Olcostephanusstephanophorous /am 0.5 5

Saynoceras verrucosum /am 9 12

Valanginites bachelardi /am 8 14

LOK.16 Baumugne Section, France, 44.60395, 5.72358;

Bulot, L., Thieuloy, J.-P., Blanc, E., 1992, Le cadre stratigraphique du Valanginiensupérior et de l’Hauterivien du Sud-Est de la France. Geologie Alpine 68:13-56; p. 25, Table V, Section spans upper Valanginian in part.

Karakaschiceraspronecostatum /am 10 11

Neocomitesneocomiensis /am 0 12

Neocomites subtenuis /am 0 12

Neocomitesteschenensis /am 0 12

Saynocerasverrucosum /am 0 4

LOK.17 LaCharceCombe Reboul, Vocontian Basin 75 km NE of Avignon, France, 44.46778, 5.4340; Bulot, 1995 dissertation; Bulot, L., Thieuloy, J.-P., Blanc, E., 1992, Le cadre stratigraphique du Valanginiensupérior et de l’Hauterivien du Sud-Est de la France. Geologie Alpine 68:13-56; Section spans middle part of upper Hauterivian Stage.

Acrioceraspulcherrinum /am 35 40

Acrioceras seringuei /am 46 46

Balearitesbalearis /am 45 49 Base B. balearis zone u.Hauterivian

Crioceratitesduvalii /am 0 4

Crioceratitesbasseae /am 45 45 ID as cf.

Crioceratitesfabreae /am 47 49

Crioceratitesremanei /am 45 45

Crioceratitesmajorisensis /am 34 42

Megacriocerasdoublieri /am 43 43

Paraspiticerasprecrassispinum /am 38 38

Plesiospitidiscusligatus /am 15 22FO = base P. ligatus Zone in upper Hauterivian

Protacriocerasornatum /am 42 44

Pseudomoutoniceras annulare /am 23 23

Subaspinocerasmulsanti /am 44 44

Subsaynella mimica /am 9 11

Subsaynellasayni /am 5 5

LOK.18 Chamaloc-Col du Rousset, France, 44.83694, 5.39944;

Bulot, L., Arnaud-Vanneau, A., Blanc, L., 1992, fig. 1-7, p. 40, Guidebook, First day: Saturday, May 9; Basin type Successions. Hauterivian-Barremian.

Base Barremian /mb 271.5 *

Balearites balearis /am 255 255

Barremites spp. /AM 246 246

Crioceratitesduvalii /am 147 180

Crioceratitesnolani /am 180 190

Neolissocerasgrasianum /am 180 180

Plesiospitidiscusligatus /am 180 180 ID as cf.

Pseudothurmanniaangulicostatum /am 263 266 ID as cf.

Pseudothurmanniacatulloi /am 266 266

Ptychophyllocerassemisulcatum /am 180 180 ID as cf.

Subsaynella sayni /am 145 180

LOK.19 Mont Aiguille I, Vercors, France , 44.84202, 5.55305;

Busnardo, Clavel, Charollais, & Schroeder, 1991, Revue de Paleobiologie, v. 10, p. 359-364; Hauterivian/Barremian.

Acrioceras tabarelli /am 266 266

Crioceratitesnolani /am 55 55sp. indet. Assoc. with Crioceratitesnolani

Emericiceras emerici /am 266 266

Pseudothurmanniapicteti /am 100 100

Sornayitesgp. simionescui /am 100 100

Plesiospitidiscusligatus /am 120 250 ID as sp.

Pseudothurmanniaangulicostatum /am 250 250 ID as group

Pseudothurmanniapseudomalbosi /am 120 120

Barremites spp. /am 291 292 group difficilis

Emericiceras emerici /am 291 292From section II so 285 m in I == 1 m in II

Orbitolinopsisflandrini /FB 304 304

Orbitolinopsissubkiliani /fb 304 344

Paleodictyoconuscuvillieri /fb 304 344

Paracoskinolinahispanica /fb 344 344 ID questioned

Paracoskinolinamaynci /fb 304 344 ID as cf.

Praedictyorbitolinacarthusiana /fb 304 344

Valserinabroennimanni /fb 304 344

LOK.20 Mont Aiguille II, Vercors, France , 44.84202, 5.55305;

Busnardo, Clavel, Charollais, & Schroeder, 1991, Revue de Paleobiologie, v. 10, p. 359-364.

Barremites spp. /am 7 8 group difficilis

Emericiceras emerici /am 7 8

Orbitolinopsisflandrini /FB 20 20

Orbitolinopsissubkiliani /fb 20 60

Paleodictyoconuscuvillieri /fb 20 60

Paracoskinolinahispanica /fb 60 ID questioned

Paracoskinolinamaynci /fb 20 60 ID as cf.

Praedictyorbitolinacarthusiana /fb 20 60

Valserinabroennimanni /fb 20 60

LOK.21 Miravetes Section, Spain, 30° 05’35”N, 1° 52’32”W;

Aguado, R., Company, M., Tavera, M., 2000, The Berriasian/Valanginian boundary in the Mediterranean region: new data from the Caravaca and Cehegín sections, SE Spain. Cretaceous Research 21:1-21, fig. 3. Base Valanginian.

Berriasella callisto /am 2.0 36.5

Erdenella paquieri /am 1.0 41.0

Fauriella boissieri /am 0.1 41.0

Kilianellagr.chamalocensis /am 7 50.0

Kilianella lucensis /am 34.4 49.0

Kilianellaroubaudiana /am 48 50.0

Kilianella roubaudi /am 47.3 49.0 spelled roubaudiana

Leptocerasstuderi /am 9.8 31.5

Neocomites premolicus /am 49.0 49.0

Neolissoceras grasianum /am 0.1 49.0

Olcostephanus drumensis /am 31.5 52.0

Spiticeras gr. multiforme /am 0.1 31.0

Thurmannicerasgratianopolitense /am 47.3 49.0

Thurmanniceras otopeta /am 31.5 41.0

Thurmanniceraspertransiens /am 47.3 52.0

Tirnovellaalpillensis /am 2.0 41.0

Calpionella alpina /ca 30.7 36.5 ID as aff

Calpionellites darderi /ca 47.3 52.0 ID questioned

Calpionellopsis oblonga /ca 30.7 52.0

Calpionellopsis simplex /ca 30.7 34.4

Lorenziella hungarica /ca 31.5 52.0

Precalpionellitesmurgeanui /ca 53 53.0

Remaniellacadischiana /ca 51.0 51.0

Remaniellafilipescui /ca 32 37.5

Remaniella murgeanui /ca 52.0 52.0 ID as PrecalpionellitesTintinnopsellacarpathica /ca 30.7 52.0

Tintinnopsella longa /ca 30.7 52.0

Calcicalathinapraeoblongata /nn 7.5 49.0

Percivalia fenestrata /nn 18.5 50.0

Percivalia nebulosa /nn 2.0 45.0

Rucinolithus wisei /nn 4.0 51.0

Speetoniacolligata /nn 10.0 50.0

Tubodiscusjurapelagicus /nn 7.0 46.0

Tubodiscus verenae /nn 18.0 50.0

LOK.24 Barlya Section, Bulgaria; 43.10116, 22.9848;

Lakova, I., Grabowski, J., Stoykova, K., Petrova, S., Rehakova, D., Sobien, K., Schnabl, P. 2017, Direct correlation of Tithonian/Berriasian boundary calpionellid and calcareous nannofossil events in the frame of magnetostratigraphy:new results from the West Balkan Mts, Bulgaria, and review of existing data. GeologicaBalanica 46:47-56, Fig. 1. Lakova, I., Stoykova, K., Ivanova, D., 1997, Tithonian to Valanginianbioevents and integrated zonation on calpionellids, calcareous nannofossils and calcareous dinocysts from the Western Balcanides, Bulgaria. MineraliaSlovaca 29:301-303; **Magnetochrons.**

Calpionella alpina /CA 45 75

Calpionellaelliptalpina /CA 50 50

Calpionella elliptica /ca 70 70

Calpionellagrandalpina /CA 45 50

Calpionellitesdarderi /CA 100 130

Calpionellitesmajor /ca 115 130

Calpionellopsis oblonga /CA 80 110

Calpionellopsis simplex /CA 75 85

Chitinoidellaboneti /ca 35 35

Chitinoidelladobeni /ca 30 30

Praetintinnopsellaandrusovi /ca 40 40

Remaniella ferasini /ca 65 65

Remaniellamurgeanui /ca 95 110 In Praecalpionellites??

Tintinnopsellacarpathica /ca 40 180

Tintinnopsella longa /ca 70 130

Tintinnopsellaremanei /ca 40 40

Assipetrainfracretacea /nn 60 145

Calcicalathinaoblongata /nn 150 165

Conusphaera mexicana /nn 19 150

Conusphaera mexicana minor /nn 17 40

Cruciellipsiscuvillieri /nn 50 180

Diadorhombus rectus /nn 170 175

Faviconusmulitcolumnatus /nn 19 60

Micrantholithusspeetonensis /nn 80 180

Microstauruschiastius /nn 45 160

Nannoconus bermudezii /nn 155 175

Nannoconus cornutus /nn 140 180

Nannoconus globulus /nn 60 80

Nannoconus quadratus /nn 140 165

Nannoconus steinmannii /nn 80 180

Nannoconus steinmannii minor /nn 75 125

Polycostella beckmannii /nn 20 50

Polycostella senaria /nn 30 50

Tubodiscus verenae /nn 145 165

Umbria granulosa /nn 40 130

Carpistomiosphaeraborzai /dn 0 17

Carpistomiosphaeratithonica /dn 17 20

Carpistomiosphaeravalanginiana /dn 130 165

Colomisphaeracieszynica /dn 30 70

Colomisphaeraconferta /dn 100 180

Colomisphaerafortis /dn 40 70

Colomisphaerahelicosphaera /dn 120 175

Colomisphaera nagyi /dn 0 17

Colomisphaerapieniniensis /dn 0 17

Colomisphaeratenuis /dn 30 75

Colomisphaeravolgeri /dn 130 180

Parastomiosphaeramalmica /dn 20 30

Stomiosphaeraechinata /dn 175 180

Stomiosphaeraproxima /dn 45 90

Stomiosphaerawanneri /dn 95 165

LOK.25 Gyangze Section, Tibet, N28° 51’85”, E89° 49’36”;

Zhang, Y., Gao, L., Wan, X., 2010, Late Jurassic-Early Cretaceous marine stratigraphy in southern Tibet. Lethaia, 43, DOI: 10.1111/j.1502-3931.2010.00238.x.Jiabula valley 20 km east of Gyangze, Tibet.

Biscutumconstans /nn 60 280

Calcicalathinaoblongata /nn 120 310

Cyclagelosphaeradeflandrei /nn 60 280

Cyclagelosphaeramargerelii /nn 60 300

Cyclagelosphaeratubulata /nn 300 300

Diazomatolithuslehmanii /nn 120 300

Discorhabdus ignotus /nn 80 80

Hexalithusmagharensis /nn 100 100

Hexalithus noelae /nn 60 60

Manivitella pemmatoidea /nn 60 300

Nannoconus steinmannii /nn 60 200

Nannoconus steinmannii minor /nn 80 200

Polycostella senaria /nn 80 310

Speetonia colligata /nn 120 310

Watznaueria barnesae /nn 80 310

Watznaueria biporta /nn 60 240

Watznaueria fossacincta /nn 60 310

Watznaueriamanivitiae /nn 60 200

Watznaueriaovata /nn 60 200

Corongoceras sp. /am 40 *

Haplophyllocerasstrigile /am 40 *

Himalayites sp. /am 40 *

Spiticeras sp. /am 50 *

Inoceramuseveresti /bi 50 *

*Interpolated from Nagarze section between top of Weimei Fm. and base of Sangxiu Fm.

Marker bed Tuff 136+/-3.0 Ma /mb 220 *

LOK.26 FiumeBosso Section, Italy, 43.42699, 12.97485;

Bralower, T.J., Monechi, S., Thierstein, H.R., 1989, Calcareous nannofossil zonation of the Jurassic-Cretaceous boundary interval and correlation with the geomagnetic polarity timescale. Marine Micropaleontology 14:153-235, Fig. 3, p. 162. **Magnetochrons.**

Magnetochron M15n /ma * 387

Magnetochron M15r /ma * 380

Magnetochron M16n /MA * 375

Magnetochron M16r /MA * 363

Magnetochron M17n /MA * 355

Magnetochron M17r /MA * 349

Magnetochron M18n /MA * 328

Magnetochron M19n /ma * 320

Magnetochron M20n /ma * 306

Assipetra infracretacea /nn 338 404

Biscutum constans /nn 350 350

Braarudosphaera bigelowii /nn 385 407

Calcicalathina oblongata /nn 387 399

Conusphaera mexicana /NN 300 399

Conusphaera mexicana minor /nn 301 334

Cretarhabdusangustiforatus /nn 362 404

Cretarhabdus conicus /nn 356 356

Cretarhabdussurirellus /nn 323 407

Cruciellipsiscuvillieri /nn 328 407

Cyclagelosphaeradeflandrei /nn 300 407

Cyclagelosphaeramargerelii /nn 300 407

Diazomatolithuslehmanii /nn 310 404

Discorhabdusrotatorius /nn 350 350

Hexalithus noelae /nn 313 332

Lithraphiditescarniolensis /nn 310 407

Manivitellapemmatoidea /nn 332 404

Markaliuscircumradiatus /nn 350 370

Micrantholithushoschulzii /nn 354 407

Microstauruschiastius /nn 303 404

Nannoconus bermudezii /nn 336 372

Nannoconus broennimannii /nn 332 385

Nannoconus globulus /nn 334 372

Nannoconus infans /nn 314 368

Nannoconus dolomiticus /nn 322 354

Nannoconus kamptneri minor /nn 334 334

Nannoconus quadratus /nn 329 350

Nannoconus steinmannii /nn 329 407

Nannoconus steinmannii minor /nn 326 404

Nannoconus truitti /nn 368 368

Nannoconus wintereri /nn 323 330

Parhabdolithus asper /nn 320 399

Parhabdolithusembergeri /nn 301 407

Parhabdolithussplendens /nn 354 385

Percivalia fenestrata /nn 370 399

Pickelhaube furtiva /nn 328 332

Polycostella beckmannii /nn 301 315

Retecapsaoctofenestratus /nn 350 393 ID as Cretarhabdus

Rotelapilluslaffittei /nn 319 380

Rucinolithus wisei /nn 400 400

Umbria granulosa /nn 315 362

Vagalapilla stradneri /nn 323 323

Watznaueria barnesae /nn 300 407

Watznaueriabritannica /nn 302 400

Watznaueria communis /nn 300 404

Zygodiscus erectus /nn 313 390

LOK.27 Fonte Giordano Section, Italy, 43.42699, 12.97485;

Bralower, T.J., Monechi, S., Thierstein, H.R., 1989, Calcareous nannofossil zonation of the Jurassic-Cretaceous boundary interval and correlation with the geomagnetic polarity timescale. Marine Micropaleontology 14:153-235, Fig. 4, p. 163. Miaolica Formation: Berriasian Stage,nannofossils. **Magnetochrons.**

Magnetochron M14n /ma * 130

Magnetochron M14r /ma * 129

Magnetochron M15n /ma * 119

Magnetochron M15r /ma * 110

Magnetochron M16n /ma * 103

Magnetochron M16r /ma * 84

Magnetochron M17n /ma * 70

Magnetochron M17r /ma * 65

Magnetochron M18n /ma * 33

Magnetochron M19n /ma * 14

Assipetrainfracretacea /nn 93 131

Biscutum constans /nn 65 65

Calcicalathinaoblongata /nn 131 132

Conusphaera mexicana /nn 5 120

Conusphaera mexicana minor /nn 46 46

Cretarhabdusangustiforatus /nn 95 125

Cretarhabdusoctofenestratus /nn 76 95

Cretarhabdussurirellus /nn 40 132

Cruciellipsiscuvillieri /nn 72 132

Cyclagelosphaeradeflandrei /nn 5 132

Cyclagelosphaeramargerelii /nn 5 132

Diazomatolithuslehmanii /nn 6 132

Hexalithus noelae /nn 13 30

Lithraphiditescarniolensis /nn 16 132

Manivitellapemmatoidea /nn 123 132

Micrantholithushoschulzii /nn 99 130

Microstauruschiastius /nn 6 95

Microstaurus quadratus /nn 15 130

Nannoconus bermudezii /nn 125 125

Nannoconus dolomiticus /nn 59 59

Nannoconus steinmannii /nn 40 131

Nannoconus steinmannii minor /nn 17 127

Nannoconus wintereri /nn 40 40

Parhabdolithus asper /nn 16 132

Parhabdolithusembergeri /nn 5 132

Percivalia fenestrata /nn 125 131

Polycostellabeckmannii /nn 6 13

Rhagodiscus nebulosus /nn 100 100

Rotelapilluslaffittei /nn 40 131

Umbria granulosa /nn 10 76

Watznaueria barnesae /nn 5 132

Watznaueriabritannica /nn 59 99

Watznaueria communis /nn 5 125

LOK.28 PuertoEscaño Section, Southern Spain, 37° 27'N, 4° 17'W;

Cordoba, Betic Cordillera; Pruner, P., Houša, V., Olóriz, F., Košták, M., Krs, M., Man, O., Schnabl, P., Venhodová, D., 2010, High-resolution magnetostratigraphy and biostratigraphic zonation of the Jurassic/Cretaceous boundary strata in the Puerto Escano section (southern Spain). Cretaceous Research 31:192-206. Calpionellids, ammonites. **Magnetochrons.**

Calpionella alpina /ca 2.35 7.50

Calpionellaelliptalpina /ca 3.06 4.15

Calpionellagrandalpina /ca 2.48 4.15

Chitinoidellaboneti /ca 0.24 1.27

Chitinoidelladobeni /ca 0.1 1.14 In Longicollaria

Chitinoidellaslovenica /ca 0.1 0.1 InBorziella

Crassicollariabrevis /ca 2.15 4.01

Crassicollariacolomi /ca 2.88 4.01

Crassicollaria intermedia /ca 1.60 3.63

Crassicollariamassutiniana /ca 2.35 4.01

Crassicollariaparvula /ca 2.80 7.50

Praetintinnopsellaandrusovi /ca 1.05 1.50

Remaniella ferasini /ca 7.50 7.50

Tintinnopsellacarpathica /ca 1.40 7.50

Tintinnopselladoliphormis /ca 6.64 7.50

Tintinnopsellaremanei /ca 1.40 2.28

Magnetochron M18n /MA ** 7.6

Magnetochron M18r /MA ** 6.96

Magnetochron M19n /MA ** 5.705

Magnetochron M19n.1r /MA ** 5.585 Also known as Brodno event

Magnetochron M19r /MA ** 3.18

Magnetochron M20n /MA ** 2.58

Magnetochron M20n.1r /MA ** 1.57 Also known as Kysuca event

Magnetochron M20r /MA ** 0.105

Burckhardticerasperoni /am 0.1 0.1

Aulacosphinctessulcatus /am 0.1 2.15

Berriasellachomeracensis /am 3.4 7.5

Berriasellajacobi /am 3.4 7.5

Berriasella tithonica /am 2.3 3.3

Durangites acanthicus /am 2.3 3.3

Durangites astillerensis /am 2.3 3.3

Durangites vulgaris /am 2.3 3.3

Kutekiceraspseudocolubrinus /am 0.1 0.1

Micracanthocerasmicrocanthum /am 0.5 2.15

Moravisphinctesmoravicus /am 0.5 2.15

Oloriziceras magnum /am 0.2 0.4

Olorizicerassalariensis /am 0.2 0.4

Paraulacosphinctestransitorius /am 0.5 2.15

Protacanthodiscusberriasensis /am 3.4 7.5

Protacanthodiscusheterocosmus /am 3.4 7.5

LOK.29 Grindstone Creek, California, 39.675969, -122.534524;

Bralower, T.J., Ludwig, K.R., Obradovich, J.D., Jones, D.L., 1990, Berriasian (Early Cretaceous) radiometric ages from the Grindstone Creek Section, Sacramento Valley, California. Earth and Planetary Science Letters 98:62-73. Nannofossils and *Buchia*; zircon ages.

Assipetrainfracretacea /NN 0 140

Cretarhabdusangustiforatus /NN 40 140

Cretarhabdussurirellus /NN 0 140

Cruciellipsiscuvillieri /NN 60 130

Eiffellithus primus /NN 80 80

Grantarhabdusmeddii /NN 0 13

Micrantholithushoschulzii /NN 40 140

Microstauruschiastius /NN 0 140

Parhabdolithus asper /NN 0 140

Rhagodiscus nebulosus /NN 45 120

Rotelapilluslaffittei /NN 0 140

Buchiapacifica /BI 55 140

Buchiauncitoides /BI 0 37

Marker bed A 137 /MB 37.5 37.9

Marker bed B 137 /MB 102.5 102.9

*137.1+/-0.6 Ma, Pb/U revised to 138.46±0.29 (GTS2012:1056 Berriasian-Valanginian)

LOK.30 Miravetes-1 (Y.Mv) section, Rio Argos, Spain, 38° 05'35"N 1° 53'32"W;

Aguado, R., Company, M., Tavera, M., 2000, The Berriasian/Valanginian boundary in the Mediterranean region: new data from the Caravaca and Cehegín sections, SE Spain. Cretaceous Research 21:1-21, Fig. 3. Short Berriasian/Valanginian interval

Calcicalathinapraeoblongata /nn 7 50

Percivaliafenestrata /nn 18 51

Percivalia nebulosa /nn2.2 51

Rucinolithus wisei /nn 4 52

Speetonia colligata /nn 10 51

Tubodiscusjurapelagicus /nn 7 48

Tubodiscusverenae /nn 18 51

Umbria granulosa /nn 4 9.9

Berriasella callisto /am 2.2 38

Erdenella paquieri /am 1 42

Fauriella boissieri /am 0.1 42

Kilianellagr.chamalocensis /am 7 50

Kilianella lucensis /am 35.4 50

Kilianellaroubaudiana /am 48 50

Leptoceras studeri /am 9.9 31

Neocomites premolicus /am 50 50

Neolissocerasgrasianum /am 0.1 50

Olcostephanusdrumensis /am 32.5 53

Spiticeras gr. multiforme /am 0.1 31

Thurmannicerasgratianopolitense /am 48 50

Thurmannicerasotopeta /am 32.5 42

Thurmanniceras pertransiens /am 48 50

Tirnovella alpillensis /am 2.2 42

Calpionella alpina /ca 32 38 ID as aff.

Calpionellitesdarderi /ca 49 51

Calpionellopsis oblonga /ca 32 53

Calpionellopsis simplex /ca 32 35.4

Lorenziellahungarica /ca 32.5 53

Precalpionellitesmurgeanui /ca 53 53

Remaniellacadischiana /ca 52 52

Remaniella filipescui /ca 32 37.5

Tintinnopsellacarpathica /ca 32 53

Tintinnopsella longa /ca 32 53

LOK.31 Canada Luenga-2 (M.CL) Rio Argos, Spain, 38°03’59”N, 1°48’39”W;

Aguado, R., Company, M., Tavera, M., 2000, The Berriasian/Valanginian boundary in the Mediterranean region: new data from the Caravaca and Cehegín sections, SE Spain. Cretaceous Research 21:1-21, Fig. 4. Short Berriasian/Valanginian interval. **Magnetochrons.**

Magnetochron M14r /MA *** 9.0

Magnetochron M15n /MA *** 3.4

Magnetochron M15r /MA *** 2.1

Magnetochron M16n /MA *** 0.5

Calcicalathinapraeoblongata /nn 1.5 5.3

Percivalia fenestrata /nn 1.5 5.3

Percivalia nebulosa /nn 1.5 1.5

Rucinolithus wisei /nn 1.5 5.3

Speetonia colligata /nn 2.2 3.8

Tubodiscusjurapelagicus /nn 1.5 3.8

Berriasella callisto /am 0.1 3.0

Erdenella paquieri /am 0.6 3.0

Fauriella boissieri /am 0.4 3.0

Kilianellagr.chamalocensis /am 3.0 3.0

Kilianella lucensis /am 3.5 5.5

Kilianellaroubaudiana /am 5.1 8.2

Leptoceras studeri /am 2.0 6.5

Neocomitespremolicus /am 5.1 6.5

Neolissocerasgrasianum /am 0.4 8.2

Olcostephanus drumensis /am 2.5 8.2

Sarasinella eucyrta /am 5.5 5.7

Spiticeras gr. multiforme /am 0.4 2.0

Thurmannicerasgratianopolitense /am 5.1 6.5

Thurmanniceras otopeta /am 2.5 3.5

Thurmanniceras pertransiens /am 4.4 8.2

Tirnovella alpillensis /am 0.4 3.0

Tirnovellaromani /am 3.0 3.0

Calpionella alpina /ca 0.1 5.1 ID as aff.

Calpionellitescaravacaensis /ca 5.7 7.1

Calpionellitesdarderi /ca 4.4 7.1

Calpionellopsis oblonga /ca 0.1 6.3

Calpionellopsis simplex /ca 0.1 4.0

Lorenziellahungarica /ca 0.1 7.1

Precalpionellitesmurgeanui /ca 4.0 7.1

Remaniellaborzai /ca 5.5 5.6

Remaniellacatalanoi /ca 0.4 3.5

Remaniellacadischiana /ca 0.1 7.1

Remaniellafilipescui /ca 0.1 5.7

Tintinnopsellacarpathica /ca 0.1 7.1

Tintinnopsella longa /ca 0.1 7.1

LOK.32 Canada Luenga-3 (Y.CL_2_) Rio Argos, Spain, 38°04’05”N, 1°48’45”W;

Aguado, R., Company, M., Tavera, M., 2000, The Berriasian/Valanginian boundary in the Mediterranean region: new data from the Caravaca and Cehegín sections, SE Spain. Cretaceous Research 21:1-21, Fig. 5. Short Berriasian/Valanginian interval. **Magnetochrons.**

Magnetochron M14r /MA *** 9.5

Magnetochron M15n /MA *** 6.2

Magnetochron M15r /MA *** 4.6

Magnetochron M16n /MA *** 2.0

Calcicalathinapraeoblongata /nn 1.5 9.5

Percivaliafenestrata /nn 3.4 9.0

Percivalia nebulosa /nn 1.5 3.6

Rucinolithus wisei /nn 1.5 8.7

Speetonia colligata /nn 3.6 9.5

Tubodiscusjurapelagicus /nn 1.5 9.5

Tubodiscus verenae /nn 6.0 9.5

Berriasella callisto /am 1.8 5.1

Erdenella paquieri /am 1.8 4.6

Fauriella boissieri /am 0.7 4.7

Kilianellagr.chamalocensis /am 4.7 5.1

Kilianella lucensis /am 6.6 8.7

Kilianellaroubaudiana /am 6.6 9.5

Leptoceras studeri /am 4.3 4.3

Neocomites premolicus /am 6.6 8.2

Neolissocerasgrasianum /am 1.8 9.5

Olcostephanus drumensis /am 4.3 9.5

Sarasinella eucyrta /am 6.6 6.6

Spiticeras gr. multiforme /am 1.8 2.1

Substreblitescallomoni /am 2.1 2.1

Thurmannicerasgratianopolitense /am 6.6 9.5

Thurmannicerasotopeta /am 4.3 5.1

Thurmanniceras pertransiens /am 6.6 9.5

Tirnovella alpillensis /am 1.8 5.1

Tirnovella romani /am 5.6 5.6

Calpionella alpina /ca 0.1 6.6 ID as aff.

Calpionellitescaravacaensis /ca 8.7 9.5

Calpionellitesdarderi /ca 6.6 9.5

Calpionellopsis oblonga /ca 0.1 8.7

Calpionellopsis simplex /ca 0.1 5.1

Lorenziellahungarica /ca 0.1 9.5

Lorenzielladacica /ca 1.8 1.8

Precalpionellitesmurgeanui /ca 5.7 9.5

Remaniellaborzai /ca 4.6 6.6

Remaniellacadischiana /ca 0.1 9.5

Remaniella catalanoi /ca 0.1 2.1

Tintinnopsellacarpathica /ca 0.1 9.5

Tintinnopsella longa /ca 0.1 9.2

LOK.34 Tang-E Asbu, Kuh-E Ginau, Iran, 56.148771, 27.42478;

Edgell, H.S., 1967, Calpionellid stratigraphy and the Jurassic-Cretaceous boundary in south-east Iran. Memoire BRGM France, No. 75, p. 213-247, text-fig. 8. Calpionellids and benthic forams.

Calpionella alpina /CA 75 360

Calpionella elliptica /CA 330 455next higher base = 315;?ID at 585

Calpionellitesdarderi /CA 485 645next higher base = 470

Calpionellopsisoblonga /CA 340 595ID as Tintinnopsella /CA

Calpionellopsis simplex /CA 375 375

Remaniellacadischiana /CA 465 675ID as Tintinnopsella /CA

Stenosemellopsishispanica /CA 340 605

Tintinnopsellacarpathica /CA 275 515

Nannoconussteinmannii /NN 455 455

Pseudocyclamminalituus /FB 565 645

LOK.35 Le Chouet, Drôme, France, 44.54235, 5.55359;

Wimbledon, W.A.P., Rehákova, D., Pszczółkowski, A., Casellato, C.E., Halásová, E., Frau, C., Bulot, L.G., Grabowski, J., Sobień, K., Pruner, P., Schnabl, P., Čížková, K., 2013, An account of the bio- and magnetostratigraphy of the Upper Tithonian-Lower Berriasian interval at Le Chouet, Drôme (SE France). GeologicaCarpathica 64:437-460, figs. 4, 10, 11, 12, 18; Upper Jurassic-Lower Cretaceous calpionellids, nannofossils, ammonites.

Magnetochron M19n /MA *** 29.0 top incomplete

Magnetochron M19r /MA *** 19.9 Base between 19.9-20.5

Magnetochron M20n /MA *** 16.6

Calpionella alpina /CA 12.6 32.2

Calpionellaelliptalpina /CA 22.2 23.0

Calpionellagrandalpina /CA 16.0 27.1

Crassicollariabrevis /CA 13.6 27.2

Crassicollariacolomi /CA 17.5 30.0

Crassicollaria intermedia /CA 12.8 23.0

Crassicollariamassutiniana /CA 12.6 27.6

Crassicollariaparvula /CA 12.6 32.2

Praetintinnopsellaandrusovi /CA 13.4 13.4

Tintinnopsellacarpathica /CA 12.8 27.7

Tintinnopsellaremanei /CA 12.2 13.2

Conusphaera mexicana /NN 12.8 32.2

Conusphaera mexicana minor /NN 13.0 32.2

Cretarhabdusoctofenestratus /NN 30.2 31.0

Cretarhabdussurirellus /NN 18.0 32.0

Cruciellipsiscuvillieri /NN 25.0 32.0

Diazomatolithuslehmanii /NN 16.0 30.2

Faviconusmulticolumnatus /NN 12.8 31.0

Hexalithusgeometricus /NN 26.0 32.0

Hexalithus noelae /NN 12.8 27.6

Manivitellapemmatoidea /NN 16.6 18.0

Microstauruschiastius /NN 12.8 32.0

Microstaurus quadratus /NN 18.0 28.6

Nannoconus erbae /NN 18.0 31.0

Nannoconus globulus globulus /NN 22.0 32.2

Nannoconus globulus minor /NN 16.4 31.0

Nannoconuskamptneri minor /NN 28.2 31.0

Nannoconussteinmannii minor /NN 28.2 30.8

Nannoconus wintereri /NN 21.8 30.8

Polycostellabeckmannii /NN 16.0 29.1

Polycostella senaria /NN 26.0 28.2

Rhagodiscus asper /NN 25.1 31.0

Umbria granulosa /NN 26.0 31.0

Watznaueria britannica /NN 12.8 32.2

Zeugrhabdotusembergeri /NN 12.8 31.0

Zeugrhabdotus fluxus /NN 12.8 31.0

Zygodiscus erectus /NN 12.8 *** *ID as Zeugrhabdotus

Ardesciella rhodanica /am 19.6 19.6

Berriasellasubcallisto /am 21.2 21.8

Berriasella tithonica /am 19.6 19.6 *Generic ID in parens

Boughdiriellachouetensis/am 17.3 19.6

Busnardoicerasbusnardoi/am 18.0 19.6

Dalmasiceras biplanum /am 29.2 29.2

Dalmasicerascrassicostatum/am 23.9 23.9

Dalmasicerasdjanelidzei/am 30.5 30.5

Dalmasicerassubloevis/am 26.1 30.0

Elenaeliacularensis/am 21.2 21.2

*Mazenoticerastarini /am 20.7 21.8 *Genus in parens

Micracanthocerasmicrocanthum/am 8.3 17.0 *ID as aff.

Moravisphinctesfischeri/am 13.0 17.0

Paraulacosphinctessenoides/am 13.0 17.0

*Protancyclocerasbicostatum /am 23.9 23.9 *ID as aff.

Protacanthodiscusandreaei/am 17.0 19.6 *ID as group

Pseudoacanthodiscushexagonus/am 19.6 19.6

Spiticeraspseudogroteanum/am 23.9 29.1

Subthurmanniaoccitanica/am 21.8 21.8 *In TirnovellasensuRetowski

Boughdiriellachouetensis /am 3.7 5.9

Protacanthodiscusandreaei /am 3.3 5.9

Protacanthodiscushexagonus /am 5.9 5.9

Toucasiellagerardi /am 3.3 3.3

LOK.36 Crimea East, Dvuyakornaya Bay, Crimea, 35°22'E, 45° N;

Arkadiev et al., 2018 Cretaceous Research 87:5-41; Upper Jurassic-Lower Cretaceous calpionellids; Dvuyakornaya Formation composite section (Fig. 3); J/K estimated @280m.

Magnetochron M18n /MA *** 366

Magnetochron M18r /MA *** 320

Magnetochron M19n /MA *** 286

Magnetochron M19n.1r /MA *** 246

Magnetochron M19r /MA *** 196

Magnetochron M20n /MA *** 132

Calpionella alpina /CA 300 384

Calpionella elliptica /CA 372 372

Calpionellagrandalpina /CA 326 326

Chitinoidella boneti /CA 100 240

Chitinoidella dobeni /CA 35 35 *In Longicollaria

Crassicollaria brevis /CA 265 265 *ID as cf.

Crassicollariamassutiniana /CA 265 312

Crassicollariaparvula /CA 240 382

Praetintinnopsellaandrusovi /CA 240 240

Tintinnopsellacarpathica /CA 228 384

Tintinnopsella longa /CA 372 372

Tintinnopsellaremanei /CA 234 234

Berriasellachomeracensis /AM 286 300

Berriasellasubcallisto /AM 372 372

Paraulacosphinctestransitorius /AM 166 166

Pseudosubplaniteslorioli /AM 312 366

Pseudosubplanitesponticus /AM 366 366

LOK.37 Crimea West, Tonas River, Crimea, 34°37'E, 44° 45'N;

Arkadiev et al., 2018 Cretaceous Research 87:5-41; Upper Jurassic-Lower Cretaceous calpionellids; ammonites, magnetochrons; Dvuyakornaya Formation composite section, Tonas River Basin (Fig. 4); J/K at unconformity @ 50m.

Magnetochron M18n /MA *** 268

Calpionella alpina /CA 25 205

Calpionella elliptica /CA 135 135 *ID as aff.

Chitinoidella boneti /CA 5 5

Crassicollariaparvula /CA 45 193

Tintinnopsellacarpathica /CA 45 167

Berriasella jacobi /AM 25 25

Pseudosubplaniteslorioli /AM 193 215

Pseudosubplanitesponticus /AM 193 193 *ID as cf.

LOK.38 Leube Quarry, Salzburg, Austria, 47.717386, 13.051135;

Bujtor, L., Krische, O., Gawlick, H.-J., 2013, Late Berriasian ammonite assemblage and biostratigraphy of the Leube quarry near Salzburg (Northern Calcareous Alps, Austria). NuesJarbuchGeologischePaläontologischAbhandlungen 267/3:273-295, p. 277.

Calpionella alpina /CA -15 275

Calpionella elliptica /CA 257 290

Calpionellopsis oblonga /CA 290 335 *ID as genus

Calpionellitesdarderi /CA 325 335 *ID as genus

Crassicollaria intermedia /CA -10 58 *ID as genus

Remaniellacadischiana /CA 262 262

Remaniella ferasini /CA 87 87 *ID as genus

Berriasellapicteti /AM 315 315 *ID as sp. aff.

Fauriella boissieri /AM 300 300

Neolisocerassalinarium /AM 315 315 *ID as genus

Protetragonitesquadrisulcatus /AM 320 320

Tirnovellaalpillensis /AM 315 315

LOK.39 Guidaloca Section, NW Sicily, Italy, 38.047835, 12.84274;

Andreini, G., Caracuel, J.E., Parisi, G., 2007, Calpionellid biostratigraphy of the Upper Tithonian-U.Valanginian interval Western Sicily (Italy). Swiss Jour. Geosciences 100(2), 179-198, figs. 2, 4.

Borzaiella atava /ca 29 29

Calpionella alpina /ca 3.5 26.5

Calpionella elliptica /ca 13 25

Calpionellitescoronata /ca 41 97.5

Calpionellitesdarderi /ca 38 116.5

Calpionellitesmajor /ca 41.5 116.5

Calpionellopsis oblonga /ca 29 116.5

Calpionellopsis simplex /ca 24.5 31

Chitinoidellaboneti /ca 2.3 3.1

Chitinoidelladobeni /ca 1.95 2.3 *ID as Longicollaria

Chitinoidellaslovenica /ca 1.95 2.3 *ID as Borziella

Crassicollariabrevis /ca 3.5 7.5

Crassicollariacolomi /ca 4.5 7.5

Crassicollaria intermedia /ca 3.5 7.5

Crassicollariamassutiniana /ca 4.5 7.5

Crassicollariaparvula /ca 4.5 10.5

Dobeniella bermudezi /ca 2.3 3.1

Dobeniella cubensis /ca 2.3 3.1

Praetintinnopsellaandrusovi /ca 3.5 3.5

Remaniella borzai /ca 14.5 26

Remaniellacadischiana /ca 14.5 25.5

Remaniellacatalanoi /ca 9 17.5

Remaniella colomi /ca 17 25

Remaniella dadayi /ca 38 97.5 *ID as "Praecalpionellites"

Remanielladuranddelgai /ca 11 14.5

Remaniella ferasini /ca 9 17.5

Remaniella filipescui /ca 29 38

Remaniella murgeanui /ca 38 97.5 *ID as Praecalpionellites

Sturiella oblonga /ca 28.5 28.5

Tintinnopsellacarpathica /ca 3.5 126.5

Tintinnopsella longa /ca 25 91.5

Tintinnopsellaremanei /ca 3.5 3.5

LOK.40 Dieni I & II Sections, W Sicily, Italy, 37.680415, 12.950598;

Andreini, G., Caracuel, J.E., Parisi, G., 2007, Calpionellid biostratigraphy of the Upper Tithonian-U.Valanginian interval in Western Sicily (Italy). Swiss Jour. Geosciences 100(2), 179-198, fig. 3.

Calpionella alpina /ca 9 14.7

Calpionellitesdarderi /ca 15.4 26

Calpionellites major /ca 23.5 25.8

Calpionellopsis oblonga /ca 15.4 25.8

Crassicollariabrevis /ca 9 9.5

Crassicollaria intermedia /ca 9 9.5

Crassicollariamassutiniana /ca 9 9.5

Crassicollariaparvula /ca 9 14.7

Remaniella catalanoi /ca 10 14.5

Remaniella dadayi /ca 15.4 25.5 *ID as "Praecalpionellites"

Remanielladuranddelgai /ca 11 12.5

Remaniellaferasini /ca 14.5 14.7

Remaniella filipescui /ca 24 21

Remaniella murgeanui /ca 15.4 22.6 *ID as Praecalpionellites

Tintinnopsellacarpathica /ca 9 12.5

Tintinnopsella longa /ca 24 25

Tintinnopsellaremanei /ca 8.5 9

LOK.41 Polaveno, Italy, 45.657829, 10.128109;

Polaveno Brescia, Italy, intersection of roads SP 10 and 48. Channell, J.E.T., Cecca, F., Erba, E., 1995, Correlations of Hauterivian and Barremian (Early Cretaceous) stage boundaries to polarity chrons. Earth and Planetary Science Letters 134: 125-140, fig. 12, table 2.

Magnetochron CM3R /MB 228.0 ***

Magnetochron CM5R /MB 200.9 208.8

Magnetochron CM6R /MB 195.8 198.2

Magnetochron CM7R /MB 182.7 192.4

Magnetochron CM8R /MB 170.2 176.6

Magnetochron CM9R /MB 148.3 162.2

Magnetochron CM10 /MB 134.8 142.2

Magnetochron CM10N /MB 107.5 127.5

Magnetochron M11r /MA *** 92.8

Magnetochron M12n /MA *** 77.3

Magnetochron M12r /MA *** 58.4

Magnetochron M13n /MA *** 46.3

Magnetochron M13r /MA *** 43.9

Magnetochron M14n /MA *** 39.5

Magnetochron M14r /MA *** 32.2

Magnetochron M15n /MA *** 28.0

Magnetochron M15r /MA *** 23.0

Magnetochron M16n /MA *** 13.4

Magnetochron M16r /MA *** 10.0

Calpionellitesdarderi /ca 32.8 ***

Calcicalathina oblongata /nn 32.6 222.6

Cretarhabdusangustiforatus /nn 5.0 ***

Cruciellipsiscuvillieri /nn *** 167.5

Lithraphiditesbollii /nn 135.6 204.9

Nannoconus bucheri /nn 108.0 ***

Reinhardtitesfenestratus /nn 9.0 ***

Rucinolithusterebrodentarius /nn 173.2 ***

Tubodiscusverenae /nn 56.5 91.0

TATRACS.1 Polish Composited Sections, 19° 48.10'E, 49° 15.30'N; 38.04559, 12.86387

Grabowski, J., Pszczółkowski, A., 2006, Magnetostratigraphy and biostratigraphy of the Tithonian-Berriasian pelagic sediments in the Tatra Mountains (central Western Carpathians, Poland): sedimentary and rock magnetic changes at the Jurassic/Cretaceous boundary. Cretaceous Research 27:398-417. Andreini, G., Caracuel, J.E., Parisi, G., 2007, Calpionellidbiostratigrahy of the Upper Tithonian-Upper Valanginian interval in Western Sicily (Italy).Swiss Journal of Geosciences 100:179-198, figs. 3, 4. Composited data of three Polish outcrop sections with calpionellids and magnetochrons across Tithonian-Berriasian boundary; scale is meters in the Posrednie III Section. **Magnetochrons.**

Borzaiella atava 42.2000 42.2000

Calpionella alpina 7.5000 51.1000

Calpionella elliptica 29.1429 44.8000

Calpionellopsis oblonga 45.8000 51.1000

Calpionellopsis simplex 43.7000 49.4000

Crassicollaria brevis 7.5000 12.5000

Crassicollaria intermedia 7.5000 12.5000

Crassicollaria massutiniana 7.5000 7.5000

Crassicollaria parvula 7.5000 43.8000

Lorenziella hungarica 46.5800 49.4000

Lorenziellaplicata 16.8357 49.4000

Magnetochron M16n *** 50.0000

Magnetochron M16r *** 44.0000

Magnetochron M17n *** 42.0000

Magnetochron M17r *** 36.0000

Magnetochron M18n *** 24.0000

Magnetochron M18r *** 21.0000

Magnetochron M19n *** 18.0000

Magnetochron M19r *** 9.0000

Magnetochron M20n *** 7.0000

Magnetochron M20r *** 2.0000

Praetintinnopsella andrusovi 5.4000 5.4000

Remaniellacadischiana 34.0000 50.1000

Remaniella catalanoi 26.5714 33.4286

Remaniella colomi 25.2857 43.4000

Remanielladuranddelgai 25.2857 25.2857

Tintinnopsella carpathica 9.0000 51.1000

Tintinnopsella longa 48.0000 48.0000

**Three Sections comprising TATRACS.1:** Tatra.1 Posrednie III Section

Kryta Valley, Western Tatra Mountains, Poland, 19°48.10'E, 49°15.30'N; Grabowski &Pszczolkowski, 2006, Cret. Res. 27:398-417, Fig. 4.Jasenina Fm. 0-14m; Osnica Fm. 14-45m; Koscieliska Marl 45-50m top section.Tithonian-Berriasian boundary at LO C. brevis & C. intermedia at 12.5m.

Calpionella alpina /ca 7.5 49

Calpionella elliptica /ca 34 44.8

Calpionellopsis oblonga /ca 45.8 49

Calpionellopsis simplex /ca 44.8 49

Colomisphaeracarpathica /ca 0.8 9.5

Committosphaera pulla /ca 0.8 0.8

Crassicollariabrevis /ca 7.5 12.5

Crassicollaria intermedia /ca 7.5 12.5

Crassicollariamassutiniana /ca 7.5 7.5

Crassicollariaparvula /ca 7.5 28

Lorenziella hungarica /ca 48 49

Lorenziella plicata /ca 38.5 44.8

Praetintinnopsellaandrusovi /ca 5.4 5.4

Remaniellacadischiana /ca 34 49

Stomiosphaeramoluccana /ca 0.8 0.8

Tintinnopsellacarpathica /ca 9 48

Tintinnopsella longa /ca 48 48

Magnetochron M16n /mb * 50

Magnetochron M16r /mb * 44

Magnetochron M17n /mb * 42

Magnetochron M17r /mb * 36

Magnetochron M18n /mb * 24

Magnetochron M18r /mb * 21

Magnetochron M19n /mb * 18

Magnetochron M19r /mb * 9

Magnetochron M20n /mb * 7

Magnetochron M20r /mb * 2

Tatra.2 Posrednie II Section

Kryta Valley, Western Tatra Mountains, Poland, 19°48.15'E, 49°15.30'N;

Grabowski &Pszczolkowski, 2006, Cret. Res. 27:398-417, Fig. 5. Osnica Fm. 0-29m top section.

Borzaiella atava /ca 25.5 25.5

Calpionella alpina /ca 0.1 28.5

Calpionellaelliptica /ca 13 28.5

Crassicollariaparvula /ca 0.1 20

Lorenziella plicata /ca 1.1 28.5

Remaniellacadischiana /ca 19.5 28.5

Remaniellacatalanoi /ca 10 18

Remaniella colomi /ca 8.5 28.5

Remanielladuranddelgai /ca 8.5 8.5

Tintinnopsellacarpathica /ca 0.1 28.5

Tintinnopsellasubacuta /ca 18 18

Magnetochron M16r /mb * 30

Magnetochron M17n /mb * 25

Magnetochron M17r /mb * 21

Magnetochron M18n /mb * 7

Magnetochron M18r /mb * 4.5

Tatra.3 Rowienka Section

Lejowa Valley, Western Tatra Mountains, Poland, 19°51'E, 49°16.45'N; Grabowski &Pszczolkowski, 2006, Cret. Res. 27:398-417, Fig. 6. Osnica Fm. 0-9.5m; Koscieliska Marl Fm. 9.5-38m top section.

Borzalella atava /ca 1.5 2.0

Calpionella alpina /ca 0 38

Calpionella elliptica /ca 0 6

Calpionellopsis oblonga /ca 12 38

Calpionellopsis simplex /ca 1 29.5

Crassicollariaparvula /ca 0 1.5

Lorenziella decica /ca 0.5 0.5

Lorenziella hungarica /ca 15.4 29.5

Lorenziella plicata /ca 0.5 29.5

Praecalpionellitesfilipescui /ca 15.4 20

Praecalpionellitesmurgeanui /ca 29.5 29.5

Remaniellacadischiana /ca 0 33

Remaniella colomi /ca 3.5 3.5

Sturiella oblonga /ca 15.4 15.4

Tintinnopsellacarpathica /ca 0 38

Magnetochron M16n /mb * 32

Magnetochron M16r /mb * 2

Note: Schnabl et al. (2015) recommended data section to be in the paleogeographic area of western Tethys (Morocco, Tunisia, **Iberia, France, Italy, Central Europe**, Turkey, **Bulgaria**, **Ukraine-Crimea**, Caucasus, **western Atlantic Tethys** (Cuba, **Mexico**, **California**), eastern Tethys (**Iran**, **Tibet**, Australasia, Russian Far East, Japan), Gondwana (Iraq, Yemen, Madagascar, Argentina, Chile); bold nations indicate sections used to compile LOK2016CS.

**Table2. ProcessedTaxon Range Data of LOK2016CSDB - 10/11/2018.**

Numerical ages of first/last appearance datums (FAD/LAD) of select Cretaceous taxa and event markers in sections listed in Table1. Some ranges are controlled by very few sections so may not yet be as long as reported elsewhere. Some species have been re-assigned to other genera since the original data was recorded and may need updating. The species concepts used by some operators may differ from current concepts so the ranges may need adjusting. Negative sign is a product of the GraphCor format relative to origin point of X/Y plot. Triple asterisks, ***, mean no data. Key taxa are Berriasian-Hauterivian zone-subzone taxa of Reboulet et al. (2018, Table 1a). Key taxa are key nannofossils of Wimbledon (2018, Fig. 2). Key calpionellid taxa of Kowal-Kasprzyk (2018, Fig. 8) and Wimbledon (2018, Fig. 1).

Acaenolithus vimineus -133.1077 -132.6852

Acanthodiscus radiatus -134.7400 -134.1955

Acanthodiscus rebouli -134.7400 -134.1636

Achomosphaera neptuni -138.9376 -133.7104

Acrioceraspulcherrinum -131.3160 -131.1940

Acrioceras seringuei -131.0476 -131.0476

Acrioceras tabarelli -131.1380 -131.1380

Aldorfia dictyotum -137.5566 -137.5566

Alvellodinium falsificum -137.2120 -136.8334

Amphizygus brooksii -135.2431 -133.0506

Amphizygusinfracretacea -134.9606 -133.6908

Amphoruladelicata -140.0945 -138.8577

Amphorulametaelliptica -142.7378 -139.3360

Ancylocerasvandenheckii -129.5119 -129.5119

Aprobolocysta eilema -131.9565 -131.2306

Apteodinium maculatum -136.6099 -134.2662

Ardesciella rhodanica -145.4200 -145.4200

Assipetrainfracretacea -144.0255 -133.2411

Aulacosphinctes sulcatus -146.0500 -145.7638

Avramidiscus kiliani -130.7420 -130.5215

Axopodorhabdus dietzmannii -138.4926 -132.4042

Balearites balearis -131.3167 -130.9737

Baronniteshirsutus -139.0077 -138.8154

Barremites spp. -131.0744 -127.0287

Ages of stage boundaries from GTS 2016

Base Albian -113.1526 ***

Base Aptian -126.3620 ***

Base Barremian -130.8000 ***

Base Hauterivian -134.7000 ***

Base Valanginian -139.4000 ***

Base Berriasian -145.7000 ***

Batioladinium gochtii -137.2120 -135.2065

Batioladinium varigranosa -137.4270 -135.5266

Berriasella bebrovensis -142.7732 -140.2929

Berriasellacallisto -141.6571 -139.0270

Berriasellachomeracensis -145.7000 -143.9195

Berriasella jacobi -145.7000 -143.9195

Berriasella malbosi -144.8319 -140.1600

Berriasellaparamacillenta -145.3050 -143.8350

Berriasella picteti -142.7484 -139.9705

Berriasella privasensis -144.0134 -142.1780

Berriasella subcallisto -145.2260 -143.8150

Berriasella tithonica -145.7429 -145.3828

Biorbiferajohnewingii -142.8841 -135.7338

Biscutum constans -143.7314 -132.3883

Biscutum ellipticum -133.7024 -133.7024

Bochianitesneocomiensis -139.1038 -135.4231

Bochianites oosteri -134.7000 -134.6754

Borzaiella atava -141.0307 -141.0307

Boughdiriellachouetensis -145.5350 -145.4200

Bourkidinium granulatum -134.2319 ***

Braarudosphaera bigelowii -140.2000 -137.7382

Braarudosphaera discula -134.9844 -133.6908

Braarudosphaera regularis -134.6173 -134.4615

Breistrofferellacastellanensis -134.7162 -134.1737

Breistrofferella varappensis *** ***

Buchia pacifica -139.9672 -134.0437

Buchiauncitoides -143.8000 -141.2216

Bukrylithus ambiguus -138.3636 -132.4042

Burckhardticeras peroni -146.0500 -146.0500

Busnardoicerasbusnardoi -145.5000 -145.4200

Busnardoites campylotoxum -138.3154 -136.9144

Busnardoites desori -138.6423 -138.0846

Busnardoitessubcampylotoxum -138.7577 -138.6423

Caddasphaerahalosa -138.3375 -135.1622

Calcicalathina oblongata -141.2629 -132.9706

Calcicalathina praeoblongata -141.5261 -138.2788

Callaiosphaeridiumasymmetricum -135.3778 -132.3883

Calpionella alpina -145.8400 -138.5318

Calpionellaelliptalpina -145.4822 -144.6213

Calpionella elliptica -143.4498 -139.8342

Calpionella grandalpina -145.7178 -144.6163

Calpionellites caravacaensis -138.4691 -138.2788

Calpionellites coronata -139.5859 -138.5269

Calpionellites darderi -139.6933 -137.3208

Calpionellites major -139.5769 -137.3208

Calpionellopsis oblonga -142.8340 -137.8100

Calpionellopsis simplex -143.1319 -138.6536

Canninginopsis colliveri -134.6784 -134.1205

Chemostrat Defined in La Charce, Vocontian Basin, France (LOK13)

Carbon peak IntraVal OAEb -137.5364 ***

Carbon peak IntraVal OAEc -136.8045 ***

CarbonpeakIntraValOAEd -133.8455 -133.7818

Carpistomiosphaeraborzai -147.6000 -146.5872

Carpistomiosphaeratithonica -146.5872 -146.4085

Carpistomiosphaeravalanginiana -137.3208 -134.9104

Cassiculosphaeridiareticulata -134.7799 -133.4708

Cerbiatabulata -134.1205 -133.4708

Cheloniceras spp. -126.8662 -126.8662

Chiastozygus bilamellus -138.8630 -136.6682

Chiastozyguslitterarius -134.3131 -133.4642

Chiastozygus platyrhethus -134.2768 -133.4970

Chiastozygus striatus -135.3857 -132.9021

Chiastozygus tenuis -138.6574 -133.6908

Chitinoidellaboneti -146.0305 -145.5149

Chitinoidella dobeni -146.0500 -145.8128

Chitinoidella slovenica -146.0500 -145.7580

Chlamydophorellahuguoniotii -136.0280 -134.1574

Chlamydophorellamembranoidea -136.1109 -136.1315

Chlamydophorella nyei -135.5874 -132.3883

Circulodinium distinctum -142.5000 -132.3883

Clavihedbergella eocretacea -130.3474 -126.8198

Clavihedbergellasemielongata -130.3474 -126.8198

Clavithurmanniaforaticostata -140.2000 -140.0942

Colchidites spp. -128.0034 -127.1447

Colomiella mexicana -123.7201 -120.7139

Colomiella recta -123.7201 -116.0906

Colomisphaera cieszynica -145.8128 -143.4298

Colomisphaera conferta -139.3868 -133.8774

Colomisphaerafortis -145.2170 -143.4298

Colomisphaera helicosphaera -138.0094 -134.2217

Colomisphaera nagyi -147.6000 -146.5872

Colomisphaera pieniniensis -147.6000 -146.5872

Colomisphaera tenuis -145.8128 -143.1319

Colomisphaera volgeri -137.3208 -133.8774

Cometodinium? whitei -138.9255 -132.4910

Conusphaera mexicana -146.4681 -132.3883

Conusphaera mexicana minor -146.5872 -143.4128

Corollithion acutum -136.6949 -133.6578

Corollithion ellipticum -138.3636 -133.2411

Corollithiongeometricum -136.7162 -132.9478

Corollithion signum -135.7320 -135.7320

Coronifera oceanica -135.0444 -132.3883

Crassicollaria brevis -145.8000 -144.2813

Crassicollaria colomi -145.6200 -144.2813

Crassicollaria intermedia -145.8406 -144.2813

Crassicollaria massutiniana -145.8400 -144.2813

Crassicollaria parvula -145.8400 -140.3161

Crassicollaria [Tintinnopsella] remanei -146.0385 -145.2170

Crassiculosphaeridia reticulata -132.9592 -132.3883

Cretarhabdusangustiforatus -142.3865 -134.0437

Cretarhabdusconicus -142.8841 -132.3883

Cretarhabdus crenulatus -142.1200 -137.7962

Cretarhabdus loriei -135.8585 -133.4970

Cretarhabdusoctofenestratus -144.2960 -141.5254

Cretarhabdussurirellus -145.5000 -133.2411

Cribroperidinum sepimentum -135.3778 -132.3883

Cribrosphaerellaehrenbergii -135.7320 -135.7320

Crioceratites basseae -131.0720 -131.0720

Crioceratitesduvalii -132.1817 -131.4591

Crioceratites fabreae -131.0232 -130.9744

Crioceratites gr. quenstedti -132.8591 -132.1591

Crioceratites loryi -134.2817 -133.4636

Crioceratites majorisensis -131.3404 -131.1452

Crioceratites matsumotoi -131.9045 -131.7455

Crioceratites nolani -131.8126 -131.7007

Crioceratitesremanei -131.0720 -131.0720

Criohimantoceras gigas -136.3432 -134.9591

Criosarasinella furcillata -135.5636 -135.1138

Criosarasinellaheterocostata -135.5636 -135.2455

Criosarasinella mandovi -135.3091 -135.0043

Cruasiceras cruasense -132.2227 -132.1273

Crucibiscutum salebrosum -134.9844 -133.6908

Cruciellipsis chiasta -140.2000 -137.7962

Cruciellipsis cuvillieri -144.8333 -133.6908

Cruciplacolithus furtivus -135.2431 -133.7997

Cruciplacolithus salebrosus -141.2867 -138.0157

Ctenidodinium elegantulum -135.7338 -135.1583

Ctenidodinium scissum -136.1528 -136.1528

Cyclagelosphaera deflandrei -146.3010 -133.7024

Cyclagelosphaera margerelii -146.3010 -132.3883

Cyclagelosphaeratubulata -133.8571 -133.8571

Cyclonephelium distinctum -134.9843 -134.1205

Cyclonephelium hystrix -138.9376 -134.3957

Cymatiosphaeradelicatula -134.6205 -133.9077

Cymososphaeridium validum -136.3043 -130.9722

Dalmasiceras biplanum -144.3993 -144.3993

Dalmasicerascrassicostatum -144.9470 -144.9470

Dalmasicerasdalmasi -142.8600 -142.7980

Dalmasiceras djanelidzei -144.2650 -144.2650

Dalmasiceras punctatum -142.8600 -141.9299

Dalmasicerassubloevis -144.7197 -144.3167

Dapsilidinium warrenii -140.1667 -132.7719

Deshayesites oglanlensis -126.3000 -126.3000

Deshayesites weissi -127.1447 -127.0403

Diadorhombus rectus -138.9115 -133.4970

Diazomatolithus lehmanii -145.7200 -132.3883

Dicanthumhollisteri -142.8841 -132.3883

Dichotomites petschi -136.7091 -136.2318

Dichotomitesvergunnorum -135.6424 -135.4724

Dilomaplacinum -136.0561 -135.9853

Dingodinium albertii -137.6912 -133.7104

Dingodinium cerviculum -140.1667 -132.3883

Dingodinium europaeum -129.4191 -128.0498

Discorhabdus biradiatus -134.1413 -133.9984

Discorhabdus ignotus -142.9086 -132.3883

Discorhabdus rotatorius -142.7104 -133.6908

Discorsia nanna -134.1574 -134.1574

Dobeniella bermudezi -145.9159 -145.6432

Dobeniella cubensis -145.9159 -145.6432

Druggidiumapicopaucicum -139.3360 -131.9565

Druggidium deflandrei -137.9471 -127.9686

Druggidiumrhabdoreticulatum -137.2180 -129.3843

Durangites acanthicus -145.7429 -145.3828

Durangitesastillerensis -145.7429 -145.3828

Durangites vulgaris -145.7429 -145.3828

Eiffellithus primus -138.2250 -138.2250

Eiffellithus windi -134.9844 -134.1975

Elenaeliacularensis -145.2260 -145.2260

Eleniceras nikolovi -134.6576 -134.6250

Elenicerastchechitevi -135.4683 -135.4683

Elenicerastranssylvanicum -134.8636 -134.6727

Emericiceras emerici -131.1380 -131.0643

Endoscrinium campanula -142.5000 -132.3883

Endoscrinium glabra -136.8475 -136.8334

Erdenella paquieri -141.4619 -138.6995

Erdenella zianidia -141.8307 -141.0866

Escharisphaeridia pocockii -142.8841 -142.8841

Ethmorhabdus gallicus -139.1510 -133.6908

Ethmorhabdushauterivianus -132.9706 -132.9706

Euptychoceras meyrati -132.8591 -132.1591

Exiguisphaera phragma -137.4270 -135.5874

Fauriella boissieri -141.9948 -139.0270

Fauriella donzei -140.2000 -140.1423

Fauriella gallica -142.7980 -140.9378

Fauriella kiliani -140.1615 -138.1346

Fauriella rarefurcata -141.2354 -139.9705

Faviconusmulitcolumnatus -146.4681 -144.0255

Flabellites oblonga -134.3435 -133.7595

Foucheria modesta -140.0945 -136.6659

Fromeaamphora -135.3897 -135.3897

Gaarderella granulifera -132.7468 -132.4042

Glob'oides algeriana -127.2958 -126.8631

Glob'oides aptiense -128.0034 -127.6553

Glob'oides blowi -128.5836 -126.8198

Glob'oides gottisi -135.9654 -126.8198

Glob'oidesmaridalensis -126.8198 -124.4990

Globuligerina hoterivica -138.3380 -126.8662

Gonyaulacysta helicoidea -144.8049 -132.3883

Gonyaulacysta kostromiensis -136.8334 -133.5453

Grantarhabdus meddii -143.8000 -133.2411

Gubkinellagraysonensis -135.4723 -126.8662

Haplophylloceras strigile -144.5543 ***

Haqiuscircumradiatus -142.9390 -132.3883

Hayesitesatlanticus -135.3857 -133.9984

Hayesitesradiatus -134.9987 -132.4591

Hedbergella aptiana -130.3474 -126.8198

Hedbergella aptica -138.3380 -126.8198

Hedbergella delrioensis -135.5587 -126.8198

Hedbergella excelsa -126.8662 -126.8662

Hedbergella kuznetsovae -130.3474 -126.8662

Hedbergella sigali -138.3380 -126.8662

Hedbergella similis -130.5215 -126.8198

Heinzia [Gerhardtia] provincialis -129.5119 -129.5119

Heinziasartousi -128.1891 -128.1078

Heleneachiastius -142.6098 -132.3883

Heterosphaeridium? galliciae -136.1238 -134.1205

Hexalithusgeometricus -144.7300 -144.1100

Hexalithusmagharensis -142.0857 -142.0857

Hexalithus noelae -145.7600 -143.6528

Himantocerastrinodosum -136.3450 -134.5773

Holcodiscuscaillaudianus -130.2082 -129.8717

Holcophylloceras calypso -145.3050 -138.8346

Hystrichodinium furcatum -137.2180 -133.5453

Hystrichodiniumpulchrum -139.1087 -133.4708

Hystrichodinium voigtii -142.5000 -132.7719

Inoceramus everesti -144.1429 ***

Jeanthieuloyitesquinquestriatus -135.7342 -134.6727

Karakaschicerasbiasalense -138.3436 -138.3436

Karakaschiceraspronecostatum -137.4356 -136.7725

Kilianella busnardoi -142.0291 -141.8307

Kilianellagr.chamalocensis -141.2335 -138.4833

Kilianellalucensis -139.8947 -138.3538

Kilianella pexiptycha -140.1712 -139.1867

Kilianellaretrocostata -140.1193 -137.6375

Kilianella roubaudi -139.0159 -137.7731

Kilianellaroubaudiana -138.6767 -138.1886

Kiokansium polypes -139.3360 -132.3883

Kleithriasphaeridiumcorrugatum -137.6912 -137.6912

Kleithriasphaeridiumeoinodes -134.1313 -133.5200

Kleithriasphaeridiumfasciatum -137.6469 -133.5200

Kleithriasphaeridiumsimplicispinum -137.5442 -136.8475

Kutekiceraspseudocolubrinus -146.0500 -146.0500

Leopoldialeopoldina -134.3545 -134.1955

Leptoceras studeri -141.1232 -138.3768

Leupoldina cabri -126.8198 -126.8198

Leupoldina pustulans -130.3474 -126.8198

Leymeriellaschrammeni anterior -113.1000 -113.1000

Lithastrinusseptentrionalis -135.2431 -132.3883

Lithraphidites bollii -136.2594 -133.3817

Lithraphiditescarniolensis -145.3057 -132.3883

Lorenziella hungarica -142.3417 -138.1933

Lorenziella plicata -140.3267 -140.0733

Luppovella superba -138.9308 -138.7577

Lyticocerasnodosoplicatum -133.0500 -132.8273

Lytoceras juileti -138.8923 -138.4308

Comparison of ages by:**Bases** in Tominaga & Sager, 2010/**Tops** in Ogg et al., 2016, Fig. 13.4

Magnetochron CM0R 125.0/126.3 -126.3000 ***

Magnetochron CM3R 128.3/129.2 -132.8761 ***

Magnetochron CM5R 130.6/131.5 -133.7254 -133.4778

Magnetochron CM6R 131.2/131.9 -133.8852 -133.8100

Magnetochron CM7R 131.6/132.2 -134.2958 -133.9918

Magnetochron CM8R 132.3/132.8 -134.6876 -134.4870

Magnetochron CM9R 133.0/133.3 -135.3739 -134.9383

Magnetochron CM10n ***/133.6 -135.7970 -135.5651

Magnetochron CM10N 134.1/134.2 -136.6526 -136.0258

Magnetochron M11r 135.5/135.9 *** -137.1133

Magnetochron M12n 136.2/136.8 *** -137.5991

Magnetochron M12r 136.5/137.0 *** -138.1914

**Magnetochron M13n 137.9/138.2 *** -138.5706**

Magnetochron M13r 138.0/138.4 *** -138.6459

Magnetochron M14n 138.4/138.6 *** -138.7838

Magnetochron M14r 138.6/138.9 *** -139.0491

Magnetochron M15n 139.1/139.6 *** -139.6000

Magnetochron M15r 139.5/139.9 *** -139.7200

Magnetochron M16n 139.9/140.4 *** -141.2286

Magnetochron M16r 141.8/141.6 *** -141.7730

Magnetochron M17n 142.3/142.2 *** -142.5000

Magnetochron M17r 142.7/142.5 *** -142.7524

Magnetochron M18n 144.1/144.0 *** -143.8646

Magnetochron M18r 144.6/144.6 *** -144.2159

Magnetochron M19n 145.2/145.0 *** -144.4098

Magnetochron M19n.1r ***/145.2 *** -144.5446

Magnetochron M19r 145.4/146.3 *** -145.4050

Magnetochron M20n 146.2/146.5 *** -145.5700

Magnetochron M20n.1r ***/147.0 *** -145.8439

Magnetochron M20r 146.9/147.8 *** -146.0488

Manivitella pemmatoidea -145.5700 -132.3883

Markaliuscircumradiatus -142.7104 -137.7962

Angles section, Vocontian Basin, France (LOK11)

Marker bed 305 -137.7465 ***

Marker bed 321 -137.3399 ***

Lithostratigraphic/sequence stratigraphic contact Texas

Marker bed Al SB WA 1 -111.9000 -111.9000

Marker bed Ap SB SL 1 -127.7285 -127.7285

Volcanic tuff beds in Grindstone Creek, CA, dated between 138.7-136.5 Ma

Marker bed B 137.1 -136.6570 -136.6292

Marker bed Tuff 136 -137.1486 ***

Mazenoticeras [Malbosiceras] paramimounum -142.3533 -140.9378

Mazenoticeras tarini -145.2777 -145.1640

Megacrioceras doublieri -131.1208 -131.1208

Meiourogonyaulax pertusa pertusa -136.9617 -131.9565

Meiourogonyaulax stoveri -134.2319 -127.9106

Micracanthocerasmicrocanthum -145.9942 -145.5500

Micrantholithus hoschulzii -142.5421 -133.2411

Micrantholithus obtusus -141.1400 -132.9067

Micrantholithus speetonensis -142.8340 -133.8774

Microcalamoidesdiversus -123.7201 ***

Microhedbergella renilaevis -113.1400 -113.1400

Microstaurus chiastius -146.0024 -133.6908

Microstaurus quadratus -145.5000 -133.6908

Miculainfracretacea -141.1400 -137.7962

Moravisphinctes fischeri -145.7500 -145.5500

Moravisphinctes moravicus -145.9942 -145.7638

Muderongiaextensiva -136.4166 -136.3461

Muderongia perforata -135.5874 -134.5662

Muderongia simplex -142.7341 -134.5662

Muderongia simplex microperforata -137.4270 ***

Muderongia staurota -135.6176 -129.0826

Nannoconus bermudezii -143.4409 -132.3883

Nannoconus boletus -135.0786 -135.0786

Nannoconus bonetii -135.9922 -135.9922

Nannoconusbroennimannii -144.4829 -133.7024

Nannoconus bucheri -136.6728 -132.3883

Nannoconus colomii -144.8049 -132.3883

Nannoconuscornutus -138.8711 -133.7024

Nannoconusdolomiticus -144.2280 -142.5421

Nannoconus elongatus -135.9063 -134.2609

Nannoconus erbae -145.5000 -144.2133

Nannoconus globulus globulus -145.1433 -144.0893

Nannoconus globulus minor -145.5800 -144.2133

Nannoconus infans -144.9076 -141.6102

Nannoconuskp. kamptneri -135.9063 -133.6908

Nannoconuskamptneri minor -144.5027 -143.5469

Nannoconus quadratus -143.8117 -134.9104

Nannoconusst.steinmannii -144.5305 -133.2400

Nannoconussteinmannii minor -144.5027 -137.8855

Nannoconus truitti -141.6102 -133.2411

Nannoconus wassallii -134.9248 -126.4076

Nannoconus wintereri -145.1640 -143.6213

Neocomitescallidiscus -134.9591 -134.8636

Neocomitesflucticulus -134.7364 -134.1955

Neocomitesneocomiensiformis -137.5682 -137.1545

Neocomitesneocomiensis -139.4992 -137.1286

Neocomitespachydicranus -135.5636 -134.1955

Neocomites peregrinus -137.1940 -136.2318

Neocomitesplatycostatus -137.7465 -137.5682

Neocomites polygonius -135.1182 -135.1182

Neocomitespremolicus -139.7000 -138.3768

Neocomitessubquadratus -138.5865 -137.6086

Neocomites subtenuis -138.0550 -137.1286

Neocomitesteschenensis -138.8346 -136.1045

Neocosmoceras sayni -143.9638 -143.9390

Neohoploceras depereti -137.4236 -136.5182

Neohoploceras provinciale -138.2214 -137.6086

Neohoploceras submartini -137.7465 -137.2818

Neolisoceras salinarium -139.1615 -138.2385

Neolissoceras grasianum -141.4962 -131.8126

Occisucysta tentorium -138.8651 -136.0280

Octopodorhabdusdecussatus -138.8333 -138.0157

Octopodorhabdus polytretus -134.8708 -133.7243

Octopodorhabdus reinhardtii -136.6248 -135.8529

Odontochitina operculata -135.3778 -127.9106

Olcostephanusbalestrai -136.8291 -135.2773

Olcostephanus densicostatus -135.5318 -134.1737

Olcostephanus drumensis -140.3522 -138.1886

Olcostephanus guebhardi -138.3154 -138.2385

Olcostephanushispanicus -134.6754 -134.6754

Olcostephanus jeannoti -134.3063 -133.0500

Olcostephanus josephinus -139.0077 -138.8154

Olcostephanusnicklesi -136.3450 -135.8802

Olcostephanus sayni -134.0783 -133.3682

Olcostephanusstephanophorous -139.0077 -137.7943

Olcostephanustenuituberculatus -138.8923 -133.2606

Olcostephanusthieuloyi -135.6247 -135.4423

Olcostephanus variegatus -133.0818 -132.9227

Oligosphaeridium complex -137.7999 -132.3883

Oligosphaeridium dividuum -135.1583 -134.2319

Oligosphaeridiumpulcherrimum -138.2144 -133.7165

Oligosphaeridium verrucosum -133.5200 ***

Oloriziceras magnum -146.0360 -146.0081

Oloriziceras salariensis -146.0360 -146.0081

Oosterella cultrata -135.4045 -134.8766

Oosterellacultrataeformis -135.6591 -134.8831

Oosterella fascigera -137.0115 -136.1045

Oosterella garciae -135.9202 -134.9591

Oosterella stevenini -136.1364 -135.4045

Orbitolinopsis flandrini -131.0329 -131.0303

Orbitolinopsis subkiliani -131.0329 -130.9170

Paleodictyoconuscuvillieri -131.0329 -130.9170

Paracoskinolina hispanica -130.9170 -130.9170

Paracoskinolina maynci -131.0329 -130.9170

Paraspiticerasprecrassispinum -131.2428 -131.2428

Parastomiosphaeramalmica -146.4085 -145.8128

Paraulacosphinctes senoides -145.7500 -145.5500

Paraulacosphinctestransitorius -145.9942 -145.7638

Pareodiniaceratophora -144.1537 -135.5266

Parhabdolithus achlyostaurion -141.0347 -138.8630

Parhabdolithus asper -144.3524 -134.0437

Parhabdolithus embergeri -146.2014 -137.7382

Parhabdolithus infinitus -137.8512 -132.3883

Parhabdolithus judithae -138.2316 -137.1429

Parhabdolithus splendens -142.5421 -137.7962

Parhabdolithus swinnertonii -138.8630 -135.2842

Percivaliafenestrata -141.5231 -133.2411

Percivalia nebulosa -141.5261 -138.3867

Phoberocysta neocomica -144.8049 -132.7719

Phoberocysta tabulata -138.1201 -138.1201

Phyllocerastethys -139.0462 -138.8154

Pickelhaube furtiva -143.8646 -133.6908

Platylenticerascardioceroides -139.0077 -138.9500

Platylenticeras occidentale -139.3154 -138.8923

Plesiospitidiscusligatus -131.8126 -130.9500

Plesiospitidiscussubdifficilis -131.1691 -130.9968

Podorhabdus dietzmanni -139.3154 -138.3346

Polycostella beckmannii -146.4085 -144.4097

Polycostella senaria -145.8128 -133.4457

Polygonifera evittii -144.1537 -144.1537

Polypodorhabus madingleyensis -144.7098 -134.8731

Praedictyorbitolinacarthusiana -131.0329 -130.9170

Praetintinnopsellaandrusovi -145.9174 -145.2170

Precalpionellitesmurgeanui -139.1922 -138.1933

Prediscosphaera columnata -113.1000 -113.1000

Protacanthodiscus andreaei -145.5500 -145.4200

Protacanthodiscusberriasensis -145.3461 -143.9195

Protacanthodiscusheterocosmus -145.3461 -143.9195

Protacrioceras ornatum -131.1452 -131.0964

Protacrioceraspuzosianum -133.0500 -133.0182

Protancyloceras punicum -140.2000 -140.1615

Protetragonitesquadrisulcatus -139.7000 -138.4308

Protoellipsodiniumseghire -134.6784 -134.3957

Protoellipsodiniumtouile -135.0452 -134.2319

Pseudoacanthodiscushexagonus -145.4200 -145.4200

Pseudoceratium anaphrissum -129.3147 -128.0266

Pseudoceratium pelliferum -142.7378 -128.0498

Pseudocyclammina lituus -139.3025 -138.9158

Pseudomoutoniceras annulare -131.6088 -131.6088

Pseudosaynella termieri -126.8662 -126.8662

Pseudosubplanites euxinus -145.1543 -144.9063

Pseudosubplanites lorioli -145.2039 -144.2118

Pseudosubplanites ponticus -145.5016 -144.6087

Pseudothurmanniaangulicostatum -131.1833 -130.8507

Also as Crioceratites (Reboulet et al. 2018)

Pseudothurmanniacatulloi -130.9722 -130.7884

Pseudothurmannia ohmi -131.1076 -130.7652

Pseudothurmanniapicteti -131.6083 -131.6083

Pseudothurmanniapseudomalbosi -131.5517 -131.5517

Pterospermella aureolata -138.4684 -133.5453

Pterospermella australiensis -138.0274 -137.8201

Ptychophyllocerasdiphyllum-137.7400 -135.4724

Ptychophyllocerassemisulcatum -143.8350 -131.8126

Reinhardtites elegans -135.3857 -133.8353

Reinhardtites fenestratus -141.8957 -132.3883

Remaniella borzai -143.2230 -141.4843

Remaniellacadischiana -145.2535 -138.2788

Remaniella catalanoi -144.6541 -138.7089

Remaniella colomi -142.8450 -141.6355

Remaniella dadayi -139.6700 -138.0788

Remanielladuranddelgai -143.7521 -142.3466

Remaniella [Calpionella] ferasini -144.0545 -140.3161

Remaniella filipescui -141.6057 -138.4654

Remaniella [Calpionellopsis] murgeanui -139.7311 -138.5357

Retecapsaangustiforata -144.4756 -132.3883

Retecapsa levis -132.7582 -132.6372

Retecapsaneocomiana -143.0488 -135.5811

Retecapsaoctofenestratus -142.7104 -138.4255

Rhabdolekiskus parallelus -135.2431 -132.7582

Rhabdolithus rectus -140.0654 -137.7962

Rhagodiscus angustus -134.3004 -134.3004

Rhagodiscus asper -144.8049 -132.3883

Rhagodiscus eboracensis -136.9285 -136.9285

Rhagodiscus nebulosus -141.3879 -135.4375

Rhagodiscusreightonensis -135.5870 -133.8353

Rhagodiscus splendens -142.9720 -132.3883

Rhynchodiniopsis aptiana -134.3957 -133.5200

Rhynchodiniopsis fimbriata -137.4270 ***

Rodighieroitesrutimeyeri -136.8364 -136.5182

Rotelapillus laffittei -144.4100 -132.3883

Rotelapillus radians -143.3817 -143.3817

Rucinolithus irregularis -133.1077 -132.5869

Rucinolithusterebrodentarius -134.5935 -133.1077

Rucinolithus wisei -141.5261 -135.2186

Sarasinella biformis -137.7465 -137.6508

Sarasinella eucyrta -139.0385 -138.4654

Sarasinellahirticula -136.5182 -136.1045

Sarasinella trezanensis -138.7673 -138.3380

Saynellaclypeiformis -132.9864 -132.9227

Saynoceras verrucosum -137.9115 -137.4043

Scriniodinium attadalense -137.5543 -132.3883

Sirmiodinium grossii -135.0575 -135.0575

Sollasites horticus -141.7347 -133.2411

Sollasites lowei -132.9021 -132.9021

Sornayitesgp. simionescui -131.6083 -131.6083

Speetonia colligata -142.0147 -133.0000

Spin ramosusmultibrevis -137.8301 -134.2319

Spiniferitesdentatus -135.0444 -132.3883

Spiniferites ramosus -137.9152 -132.3883

Spiticeras gr. multiforme -141.4962 -139.8143

Spiticeraspseudogroteanum -144.9470 -144.4097

Spitidiscusfasciger -133.3682 -132.4240

Spitidiscus gr. lorioli -134.6267 -134.1737

Spitidiscus gr. pavlowi -133.5909 -132.9864

Spitidiscus hugii -130.8000 -130.5563

Staurolithites crux -134.9844 -133.6908

Stenosemellopsis hispanica -142.7684 -139.1092

Stephanolithion laffittei -140.2000 -133.2411

Stomiosphaera echinata -134.2217 -133.8774

Stomiosphaeraproxima -144.9191 -142.2383

Stomiosphaera wanneri -139.7311 -134.9104

Stradneria crenulata -143.2244 -132.3883

Sturiella oblonga -141.1063 -141.1063

Subaspinocerasmulsanti -131.0964 -131.0964

Suboosterella heliaca -133.2409 -133.2409

Subpulchellia nicklesi -130.5679 -130.2894

Subsaynella mimica -131.9504 -131.9016

Subsaynella sayni -132.2545 -131.5227

Substreblites callomoni -141.2406 -141.1765

Subthurmannia clareti -142.9717 -142.9717

Subthurmanniafloquinensis -144.2862 -143.8894

Subthurmannia occitanica -143.1133 -141.3594

Subthurmannia patruliusi -142.9220 -142.1531

Subthurmannia subalpina -144.0878 -143.5900

Subtilisphaera perlucida -135.2020 -127.9106

Subtilisphaera senegalensis -130.5679 -127.9106

Subtilisphaera terrula -135.3778 -132.3883

Systematophora areolata -138.9376 -137.3864

Systematophora fasciculigera -138.5204 -138.5204

Systematophora palmula -142.2524 -135.5812

Systematophora silybum -136.9617 -134.2319

Tanyosphaeridium boletus -139.1087 -133.5352

Tanyosphaeridium magneticum -140.0945 -128.0498

Tanyosphaeridium salpinx -143.4329 -132.3883

Tanyosphaeridiumvariecalamus -135.2020 -132.3883

Taveridiscusoosteri -130.8000 -130.6724

Tegumentum stradneri -136.1238 -132.6029

Teschenites callidiscus -135.0043 -134.7854

Teschenitescastellanensiformis -134.7698 -134.3545

Teschenitesflucticulus -134.7400 -133.9825

Teschenitesneocomiensiformis -135.9202 -134.6754

Teschenitespachydicranus -135.5153 -133.9825

Teschenitessubflucticulus -135.1138 -134.6941

Teschenitessubpachydicranus -134.9308 -134.7638

Tetrapodorhabdus coptensis -138.3636 -132.6029

Tetrapodorhabdus decorus -138.3636 -135.5671

Thurmannicerasgratianopolitense -139.6933 -138.2788

Thurmanniceras otopeta -140.3522 -138.3606

Thurmannicerasperisphinctoides -139.4000 -139.3054

Thurmanniceras pertransiens -139.7000 -138.1886

Thurmannicerassalientum -139.3869 -139.3706

Tintinnopsella carpathica -145.8685 -133.8774

Tintinnopsella doliphormis -144.2653 -143.9195

Tintinnopsella longa -143.4298 -137.3208

Tintinnopsellaremanei -146.0385 -145.2170

Tirnovellaalpillensis -141.4163 -139.0270

Tirnovella occitanica -145.2260 -145.1640

Tirnovellapertransiens -139.4000 -137.7731

Tirnovellaromani -139.2867 -139.0270

Tranolithusgabalus -138.3636 -132.4020

Tranolithus salillium -139.1510 -134.4615

Trichodinium castanea -136.9603 -134.1111

Tubodiscusjurapelagicus -141.5261 -133.2411

Tubodiscus verenae -140.8148 -133.7385

Umbria granulosa -145.2170 -137.3208

Vagalapilla compacta -140.2000 -138.4308

Vagalapilla stradneri -144.1675 -132.3883

Valanginites bachelardi -137.9115 -137.2000

Valanginites nucleus -137.7465 -137.6508

Valserinabroennimanni -131.0329 -130.9170

Varlheideites peregrinus -136.8364 -136.2318

Vekshinella angusta -136.1315 -133.3589

Vekshinella stradneri -139.1510 -133.7024

Wallodinium cylindrica -139.3360 -132.3883

Wallodinium krutzschii -142.5000 -132.3883

Wallodinium lunua -134.4897 -133.5352

Watznaueriabarnesae -146.3010 -132.3883

Watznaueria biporta -143.7314 -132.4042

Watznaueria britannica -146.1019 -132.3883

Watznaueria communis -146.3010 -132.3883

Watznaueriafossacincta -143.7314 -133.4457

Watznaueria manivitiae -143.7314 -137.9714

Watznaueria oblonga -137.0884 -132.4910

Watznaueria ovata -143.7314 -137.9714

Watznaueriasupraretacea -135.9899 -132.4271

Wollemanniceraskeilhacki anterior -113.1000 -113.1000

Wollemanniceraskeilhackikeilhacki -113.1000 -113.1000

Zeugrhabdotusembergeri -145.7600 -132.3883

Zeugrhabdotus [Zygodiscus] erectus -145.0071 -133.5530

Zeugrhabdotus fluxus -145.7600 -144.2133

Zeugrhabdotuspseudoangustus -138.3636 -133.4970

Zeugrhabdotus trivectis -134.9844 -133.6908

Zygodiscusbicrescenticus -134.9606 -133.8100

Zygodiscusdiplogrammus -138.5775 -132.3883

Zygodiscus elegans -141.6600 -132.3883

**END**
